# An encounter with the self: A thematic and content analysis of the DMT experience from a naturalistic field study

**DOI:** 10.3389/fpsyg.2023.1083356

**Published:** 2023-03-27

**Authors:** Pascal Michael, David Luke, Oliver Robinson

**Affiliations:** ^1^Centre for Mental Health, School of Human Sciences, Old Royal Naval College, University of Greenwich, London, United Kingdom; ^2^Department of Brain Sciences, Faculty of Medicine, Centre for Psychedelic Research, Imperial College London, London, United Kingdom

**Keywords:** DMT, Dimethyltryptamine, self, disconnected consciousness, thematic analysis, content analysis, naturalistic, field study

## Abstract

**Introduction:**

*N*,*N*-Dimethyltryptamine (DMT) is an endogenous serotonergic psychedelic capable of producing radical shifts in an experience that have significant implications for consciousness and its neural correlates, especially given the “disconnected consciousness” suggested by the “breakthrough” DMT state. Its increasing usage and clinical trial indicate the growing importance of a thorough elucidation of the experience's qualitative content, over and above the phenomenological structure. This is particularly in light of the intensely pervasive effects of DMT occasions in all dimensions of the self, which are often ontologically challenging yet potentially transformative.

**Methods:**

This is the second report on the first naturalistic field study of DMT use exploring its qualitative analysis. Screened, healthy, anonymized, and experienced DMT users were observed during their non-clinical use of the drug at home (40–75-mg inhaled). In-depth semi-structured interviews, inspired by the micro-phenomenological technique, were employed immediately after their experience. This study reports on the thematic and content analysis of one major domain of the breakthrough experiences elicited, the “self”; where analyses of the “other” were previously reported. A total of 36 post-DMT experience interviews with mostly Caucasian (83%) men (eight women) of a mean of 37 years were predominantly inductively coded.

**Results:**

Invariably, profound and highly intense experiences occurred. The first overarching category comprised the onset of effects, encompassing super-ordinate themes including sensory, emotion and body, and space-time shifts; the second category comprised bodily effects, encompassing themes including pleasurable, neutral/both, and uncomfortable; the third category comprised the sensorial effects, encompassing open-eye, visual, and cross-modal and other; the fourth comprised the psychological effects, encompassing memory and language, awareness and sense of self, and time distortions; and the fifth comprised the emotional effects, encompassing positive, neither/both, and challenging experiences. Many further subthemes also illuminate the rich content of the DMT experience.

**Discussion:**

The present study provides a systematic and nuanced analysis of the content of the breakthrough DMT state pertaining to one's personal and self-referential experiences of the body, senses, psychology, and emotions. The resonances both with previous DMT studies and other types of extraordinary experiences, such as the alien abduction, shamanic and near-death experiences, are also elaborated upon. Putative neural mechanisms and their promise as a psychotherapeutic agent, especially owing to deep emotional impact, are discussed.

## Introduction

### What is DMT?

*N,N-*Dimethyltryptamine (DMT) is an indolamine and classical psychedelic, which, when exogenously administered, is capable of generating brief, yet fundamental shifts in the structure and content of consciousness. Such serotonergic psychedelics are considered to exert their effects *via* the 5-HT2A receptor, though downstream GABA, glutaminergic and dopaminergic systems may be modulatory (Halberstadt et al., 2017). One essential reason for the interest in this compound is its endogenous nature, including in human beings (Barker et al., 2012), where enzymatic co-localization for DMT synthesis (INMT and AADC) has also been evidenced in mammalian brain tissue (Dean et al., 2019). A more in-depth introduction to psychedelic DMT can be found in the opening of Michael et al. (2021) study.

The precise nature and extent of DMT's physiological functions as an endogenous amine are yet to be fully elucidated. However, mounting evidence gestures to a multitude of roles in the peripheral and central nervous system, entailing possibly fulfilling criteria for being a neurotransmitter and neuromodulator, including at the 5-HT2A site—as well as mitigation of cellular stress in nervous tissue and peripheries, *via* activation of the sigma-1 receptor (Carbonaro and Gatch, 2016; Rodrigues et al., 2019). Its implication in waking consciousness, altered states thereof such as dreaming or psychosis (Dean, 2018), or extraordinary human experiences such as the near-death state or alien abduction (Luke, 2012, 2020) are speculative.

Even greater intricacies of the compound's subjective sphere, including the entity encounter phenomenon in which there has been special interest (Luke and Spowers, 2018, 2022; Davis et al., 2020a; Michael et al., 2021), may be illuminated after reports on continuous infusions of DMT conducted (e.g., Smith, 2021) based on a pharmacokinetic model to sustain peak blood-concentrations (Gallimore and Strassman, 2016). Regarding recent enthusiasm for DMT's clinical potential, such beings may be projections of facets of the broader self with deeply archetypal characteristics, both positive and challenging—which may both be productive (Davis et al., 2020b; Lutkajtis, 2020; Michael et al., 2021) and possibly difficult feature to navigate in the therapeutic process (Hill, 2019; Michael et al., 2021; Whitfield, 2021).

### “What is it like to be” on DMT?

Sai-Halasz et al. (1958) represented the first research study with DMT in humans, using mostly 0.8-mg intramuscularly (I.M.) in 30 individuals. The report superficially listed alterations in space perception, body schema (such as depersonalization), time disturbance, loosening of associations/delusional ideation, “ego loss/amnesia”, and euphoria and anxiety. Boszormenyi and Szara (1958) and Turner and Merlis (1959) subsequently administered DMT to psychiatric inpatients, the former to 24 female inpatients (mostly with schizophrenia) with 1–1.5-mg/kg I.M. DMT, documenting similar symptomological fashion: Euphoria, giggling or liveliness; anxiety and agitation; paraesthesia, i.e., abnormal sensations; laughing, sexual ecstasy, and depersonalization; and “over-estimation of time” and thought disorder.

Based on 340 internet DMT trip-reports, Meyer (1992) conceived of experiential levels 1–2 as codifying the threshold and “interior flowing” of consciousness, followed by vivid geometric patterns which may be 2D and pulsating. Strassman et al. (1994), as part of their seminal research with 60 subjects and over 400 doses of I.V. DMT, administered their Hallucinogen Rating Scale (HRS), which was categorized into the six subsections of somaesthesia, affect, perception, cognition, volition, and intensity. Strassman et al. (2008) also conceptualized the experience as encompassing the transpersonal, invisible worlds, and the personal, which pertained to self-related experiences of a deeply individual, but also often challenging nature.

The laboratory investigation into DMT by Timmermann et al. (2019) resulted in a novel and invaluable neurophenomenological report, wherein the phenomenological structure of first-person accounts closely corresponded to third-person electroencephalographic indices, namely the “visual,” “bodily,” and “metacognitive/emotional” domains identified. The present report on the DMT breakthrough experience, which may be defined as producing very strong psychoactive effects (Davis et al., 2020a) such that an emergence of a novel environment transpires, and especially Michael et al. (2021) attention to the experients' incorporation into other worlds, have explicit relevance for the neural substrates of “disconnected consciousness”. This is described by Martial et al. (2020) as subjective states without experience of the external world, that is, internal conscious content while dislocated from the immediate environment.

### The rationale for the study

A detailed rationale for the naturalistic field study of DMT upon which this study is based is outlined in Michael et al. (2021) first qualitative analysis. In contrast to the focus in that report on entity encounters, the analysis in the present report is dedicated to the experiential domains pertaining to the s*elf*; that is, a breakthrough experience not into “other worlds” but into the inner world.

Continuing investigations into the DMT experience is necessary in light of growing popular use (Winstock et al., 2014) and interest in the administration of DMT for psychiatric purposes (e.g., Liechti and Ley, 2020; Steiner et al., 2020; D'Souza, 2021; Scheidegger, 2021). Importantly, the present study provides systematic improvements over most of the significant limitations of previous survey and laboratory research on DMT (Strassman, 2001; Cott and Rock, 2008; Timmermann et al., 2018; Lyke, 2019; Davis et al., 2020a). Improvements included utilizing only breakthrough experiences; which were in a quasi-controlled setting; immediate semi-structured interviews; which used “bracketing” inspired by the micro-phenomenological technique (Petitmengin, 2006); and qualitative vs. phenomenological analysis revealing detailed content in lieu of generic structure (Varela and Shear, 1999).

The present analysis' *self*-associated themes, particularly in the emotional domain, are especially pertinent to the compound's therapeutic capacity. Possible neural mechanisms are also explored, offering an indirect corresponding between phenomenological features and objective neural correlates. Finally, the nature and degree of DMT's mimesis of the near-death experience (NDEs; Strassman, 2001; Timmermann et al., 2018) and the potential for neurochemical contribution acts as the primary focus of Michael et al. (2023, SSRN).

## Methods

### Design

This study was a field study on DMT usage in naturalistic contexts. The researchers observed individuals taking DMT in a setting of their choice, with a vapourised dose of 40–75 mg and a mean of 54.5 mg (*SD* 9.8). This guaranteed a “breakthrough” experience characterized by entry to an immersive space and rating high subjective intensity [subjective intensity rating peak > 7 (mean 9.5) on a scale of 1–10, where 1 = normal and 10 = too altered to communicate] and was followed by a semi-structured interview when the participant reported being at 1/10 intensity.

For a complete description of the *participants and recruitment, measures and materials, procedure and anonymity*, and *analyses*, refer to the Methods section of the original report (Michael et al., 2021) derived from the naturalistic field study of DMT use upon which the present, second report is based. A summary version of the methods is presented below, including those specific to the current report.

### Participants

**Figure 1:**
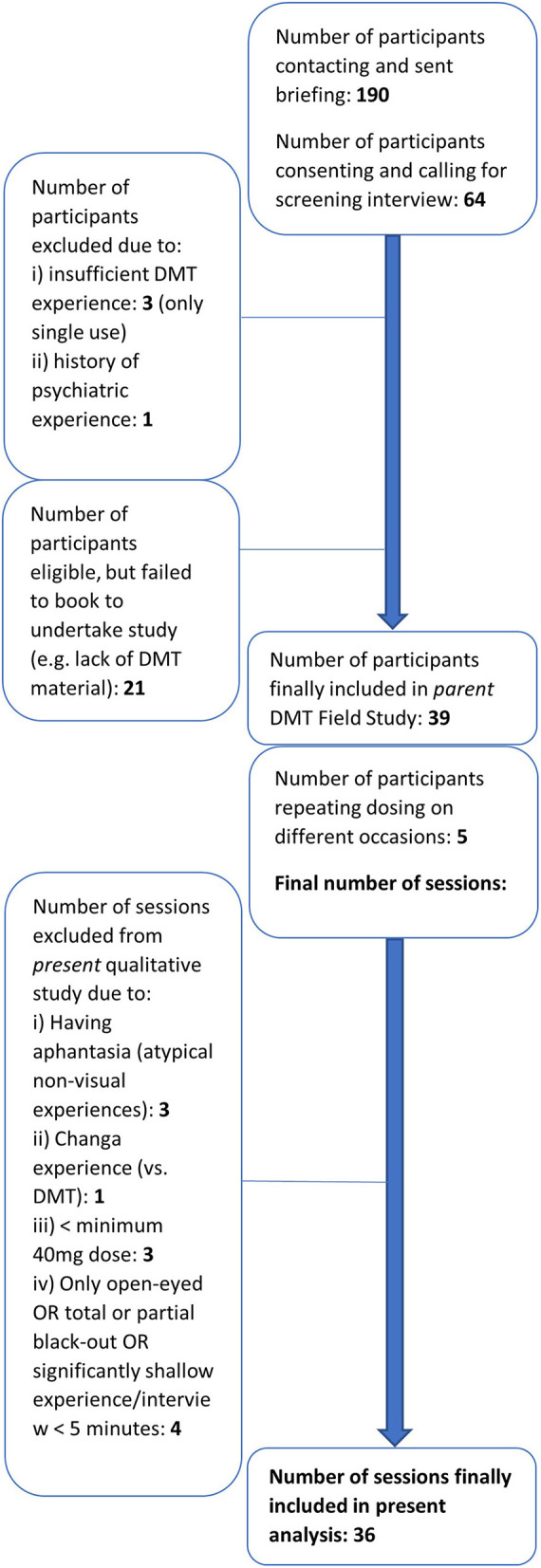
Participant recruitment flowchart—inclusion, exclusion, and final sample size overall and of the present analysis.

Volunteers were either convenience or snowball sampled, with inclusion criteria involving at least 1 breakthrough *NN-*DMT experience and other *NN-*DMT or analog experiences (see Table 1), and provided their own DMT supply—and exclusion criteria involving prior psychedelic experiences with lasting difficulties and administration of the SCID-CT (First et al., 2007) indicating current psychiatric health conditions or difficulties, or previously within a recent time frame (in line with Johnson et al., 2008). In total, 47 DMT sessions were totaled in the parent field study, with 36 sessions being the basis of the present analysis, owing to exclusion due to, for instance, reporting no memory, using changa, being classed as aphantasic (see Figure 1, for further details).

**Table 1:** Participant demographics and DMT experience.

**Participant number**	**Pseudonym**	**Age (range)**	**Sex**	**Nationality**	**First time DMT used**	**Last time DMT used**	**Overall times DMT used**	**% breakthrough DMT experiences**
1	MP	45–49	M	White British	2011	11/2016	20	33%
2	TM (3 doses)	30–34	M	White Romanian	2015	11/2016	5–6	100%
3	BB	35–39	M	White British	2013	02/2018	15–20	25%
5	JM	35–39	M	White British (Scottish)	2015	03/2017	12	66%
6	RV	40–44	M	White British	2015	08/2018	1 (+4 AYA)	75%
7	TC	25–29	M	White German	2014	06/2018	10–15	100%
8	HV	35–39	F	Black British (Ghanaian-Egyptian descent)	2016	02/2018	80	<100%
10	GR	25–29	M	White Romanian	2015	2015	2 (+ 4-ACO-DMT)	Once
11	SP (2 doses)	35–39	M	White British	2003	06/2017	10–15	>50%
12	RH (3 doses)	55–59	M	Asian British (Indian descent)	2013	08/2018	Hundreds	75%
14	AZ	25–29	M	Israeli	2013	02/2018	7	>40%
15	ZD	30–34	F	White British	2017	03/2018	20	90%
16	RS	25–29	M	Black British (African descent)	2016	05/2018	40	50%
17	LR	25–29	M	Chinese Italian (Dual)	2010	2011	25	40%
23	AF	40–44	F	White Italian	2018	05/2019	2 (+ 8 AYA, 10 changa)	>75%
24	LG	30–34	M	Mixed British (Sri Lankan-German descent)	2011	07/2019	20	20%
25	AN	25–29	F	White British	2018	07/2019	7	>40%
26	EM	20–24	F	White Romanian	2017	05/2019	10	90%
27	AB	35–39	M	White British	N/A	N/A	10	100%
30	SH	30–34	F	White British	2007	2008	6	50%
32	OR (2 doses)	25–29	M	Brazilian	2012	2018	3 (+ Hundreds AYA)	Once
34	FF	45–49	M	White British	N/A	N/A	10	80%
35	JB	40–44	M	White British	N/A	N/A	8	75%
36	BW	45–49	M	White British	2000	07/2019	3 (+ 4 changa)	Once
40	JA	35–39	M	White British	2014	10/2019	70	>70%
41	AV	45–49	F	Brazilian	2003	06/2019	20	100%
42	MS	55–59	F	Mixed British (Iraqi-Italian descent)	2013	2017	5	100%
43	DD	40–44	M	White British	N/A	N/A	Over hundred	>40%
44	DS	45–49	M	White British	N/A	N/A	Hundred	50%
47	ST	35–39	M	Nigerian	N/A	N/A	3	66%

Average age, 37.4 (range: 23–58), eight women and 22 men, 83% Caucasian (European, South American, Mixed British, Inc. Sri Lankan, Iraqi), 28% Black British, Indian British, Chinese Italian, and African); AYA, ayahuasca; N/A, unprovided (free to withhold information due to illicit activity admission).

### Data collection

A semi-structured interview (SSI) was employed immediately post-experience (see Supplementary material 4, for full interview guide), with interview probes for elaboration spanning domains including sensorial, bodily, emotional, and psychological experiences, what happened at the very start, and any encounter phenomena and visionary landscapes. The SSI most often lasted at least 30 min (approximately ranging from 12 to 75 min).

### Analysis

Interviews were audio recorded, fully transcribed, and coded *via NVivo* v.12, where the software facilitated a calculation of frequency per the content theme. Transcripts were subjected to thematic analysis using a hybrid deductive-inductive process, wherein four of the five eventual highest-order, overarching *categories* of the present report were deductively based on, but not identical to, most of the major categories dividing the HRS (including “perception,” “somaesthesia,” “affect,” “cognition”; Strassman et al., 1994). After this point, including the first category (“onset”), all analyses and coding efforts were in accordance with the guidelines as provided by Braun and Clarke (2006), as well as being purely inductive in nature with *super-ordinate themes* and *subthemes* herein elicited by the interview data only (a sixth category comprises “meta-narratives”, see Supplementary material 1, which is itself also purely inductively developed).

### Ethics

The field study and present analysis were approved by the University of Greenwich Research Ethics Committee (Ref. 17.3.5.15). Given the class-A legal classification of DMT as a controlled substance, strict anonymity of all participants was held throughout. Recruitment and data collection protocols ensured that no personal, identifiable information, such as name, phone number, or address, was shared or recorded, and all reported data were anonymized. Naturalistic field research with psychoactive substances have previously been successfully conducted (ayahuasca: Kuypers et al., 2016; psychometric/neural measures of DMT: Pallavicini et al., 2020).

## Results

The following table (Table 2) presents all levels of themes described in the present article's qualitative analysis of the DMT experience—the overarching categories comprising onset, bodily, sensorial, psychological, and emotional. This is except for final subthemes eliciting specific content, which are listed fully in Table 3 in the Supplementary material 2, where extra clarificatory notes for many of the themes can be found. Furthermore, available in the Supplementary material 3 is a graphical representation of the themes. Both in the tables and the ensuing descriptions of themes, **bold** signifies overarching categories (e.g., **onset**); *italics* signify super-ordinate themes (e.g., sensory). In the descriptions of themes only, final subthemes (e.g., “submergence”) are flanked by “apostrophes” (and a number of participant interviews in which final subthemes are present, out of total interviews, are shown in parentheses).

**Table 2:** Thematic analysis of the DMT experience: an encounter with the self; tabularization of categories, super-ordinate, and mid-level themes explored in the present article—see Supplementary Table 3, for a list of all subthemes.

**Encounter with the self**
	No. Interviews /36 (%)
**Onset: A tumultuous transition in self, space, and time**	
Sensory	14 (39)
Emotion and body	15 (42)
Space-time shifts	10 (28)
**Bodily: A blissful disembarkation from the body**	
Pleasurable	10 (28)
Neutral/both	8 (22)
Uncomfortable	9 (25)
**Sensorial: A kaleidoscopic blossoming and blending of**	
**the senses**	
Open-eye	11 (31)
Visual	27 (75)
Cross-modal and other	14 (39)
**Psychological: In inarticulable loosening of the psyche**	
Memory and language	31 (86)
Awareness and sense of self	18 (50)
Time distortions	13 (36)
**Emotional: A soaring angelic, and fathoming hell, of the soul**	
Positive	34 (94)
Neither/both	22 (61)
Challenging	6 (17)
**Meta-narratives: Co-creative ‘insight into heart and cosmos**	
Mind-manifestation	30 (83)
Ontological and emotional breakthrough	27 (75)
Transitions through time	20 (56)

### Onset: A tumultuous transition in self, space, and time

Several themes have been duplicated to appear both in their natural position (such as under sensory or psychological) and again under the category of the onset of the DMT experience, the latter representing those elements that some participants volunteered as the earliest they can recall.

#### Sensory

A total of 14 participants (39%) described significant *sensory* events at onset, such as sensations of “submergence” (9) resulting from the sheer experiential deluge at the brink of the DMT breakthrough. Thus, LR uses the terminology of there being “many, many ideas and many things coming flooding through”, and TM (trip 2) articulates that “it came like a typhoon over me”. In his third experience, he reinforces the metaphor:

“The first part happened too fast, really, too fast, the images were overflowing, I couldn't understand anything... It was like I was in the middle of a tornado, everything was going at unimaginable speed, I couldn't follow anything… maybe a sensation like I was drowned – drowning, but in colors and images”.

AN's experience was “slightly oceany in the feel” where she “felt submerged…I felt underwater in some way, or contained by an[sic] energy”. This is again redolent of ZD's submergence, who also felt she was *within* some entity:

“[it] felt like being at the bottom of the swimming pool, like hearing someone stood on the side, saying ‘how intense is the experience?’. Yeah, it felt like there were so many layers of things going on at once…that my brain could just not handle it. I was just in so many different experiences at once…

it was really chaotic, and exhausting…it felt like I was being battered around by loads of different competing currents, some of which trying[sic] to make me go deeper, and some trying to bring me out… energy streams trying to take me in different directions”.

“Geometric patterns” (6) were reported to clearly emerge around the trip's very beginning by a minority (much fewer than expected from literature or anecdote, but may be artifactual from many others not volunteering explicitly). RH (trip 2) describes this progression from colorful (basic) patterns to more iconic (complex) imagery:

“It started with softly opening up patterns, like very beautifully coloured blankets…but then all of a sudden, into the ‘death’ bit… it would start off with orange triangles, that seemed to open up [into] – would you call it geodesic? – more 3D. Then something beyond! Then out of it would come entities, out of this very fracturedness, I'd be in this completely other world[sic]”.

#### Emotion and body

Immediate sensations in both *emotion and body*, the emotions being of such intensity so as to be bodily manifest, were offered by 15 participants (42%). Some commented on a mild “fear or anxiety” (4), such as “being a scared monkey into this reality” (GR). Several subjects entered the domain of “terror or panic” (six; three of which belonged only to RH). Of important note, five of these six experiences were associated with fear, or mostly sensation, of dying (see Michael et al., 2023, SSRN, for an analysis of DMT's mimicking of the near-death experience), and many of these, in turn, concurrent with trouble breathing (potentially related to the DMT vapor). Illustratively, in the “horrifying, terrifying, horrible” moment of RH's second trip of the study, he discloses that:

“I had the terrors for a while because I was in a fairly new place I didn't really recognise, very startling... I remember fighting for breath, thought I was going to throw up at one stage… it's hard to go past the fear in the early stage, a lot of terror…

I was panicking a bit because it really felt super intense... I was really worried…that I wasn't coming back or something… dying at the beginning, pain, confusion – that really was fucking powerful actually… I seemed to be in what felt like death really”.

To further labor the theme of “labored breathing” (7), again alongside deep fear and felt a threat to life, ZD here fleshes this out:

“I wanted somebody to rescue me. Fuck, that was so horrible… I felt like I'd lost my breath or something, like a horrible feeling in my chest… I had this sense that I wasn't gonna survive it, a sense of being in [sic]existential crisis which I've never had before. God…

it was absolute panic in my heart… And I had this sense of there being something outside of me or something outside the experience that could save me, and I don't know whether that's humans – or that's something else”.

“Pain or torture” (2), ever more challenging than above but still coupled with mortal danger to ego, is illustrated by TM (trip 3) uttering that “it was a pretty tormenting experience. I didn't know what was happening, why it was happening, who am I!?... I wasn't aware of myself”.

In terms of instant alterations in bodily awareness, alluded to by TM above continuing that it was “like I was falling down”, GR was alone in expressly reporting a physical expansion (1):

“in the heart space, sort of a melty, clammy, gluey sensation, just like ^*^whoosh^*^, you know like how snow melts… Yes, it was like a small sponge [there], then got more spongey – and that transcended my body…it just started to expand beyond my parameters”.

Only a minority (though repeated in the literature) reported “the rush” sensation (5); an adrenal-like response such as the “heart…going ten to the dozen” (BB) and “heat which is rushing through everywhere” (EM), accompanied by an “acceleration feeling at the beginning…like going over a rollercoaster” (JM)—but here divorced from any panic. For example, MP simply proclaims “a kundalini hit at the base of my spine”, and TC relates, “energy is rushing from my body and kind of radiating almost”.

#### Space-time shifts

Reports pertaining to rapid *Space-time shifts* were given by 10 participants (28%), the most within experiences of “time dilation” (six; vs. 13 reporting similar distortions pervading the entire trip), such as RH's always being thrown into the throes of dying in a void-like scenario which feels “ridiculously long, like, *forever* long”. Sometimes this temporal expansion entwined with the initial inhaling, as ST continues:

“after I blew out the smoke and it reverted to normal breathing, instead of letting it go on I began to participate in it because it seemed so slowed down into so many different steps I could step in at any time”.

The dissolution of immediate surroundings, the “breakdown of reality” (4) presaging an entry into a new one, is grasped by JM's assertion that “it's very much a deconstruction feeling, things kind of peeling apart if you like”, or BW confirming a “streaming away of sort of trails…the visual field becomes less and less complex, and more bold outlines and then silhouettes then basic block colours”.

While the vast majority described a sense of some translocation from the worldly to the otherworldly (see Michael et al., 2023, SSRN, for NDE-like themes), a few insisted they were not “going through a tunnel or anything” but rather “just ^*^Bang^*^, there… arriving ‘immediately at this scene”’ (4) (MP).

### Bodily—A blissful disembarkation from the body

Participants did not generally elaborate on many experiential changes within the body, as most, in fact, stipulated feelings of disembodiment (see Michael et al., 2023, SSRN).

#### Pleasurable

The majority of statements here, by 10 participants (28%), were *pleasurable* in kind, stretching from “pain relief” (2), which can be quite striking such as TM (trip 1) divulging that “because of fibromyalgia, I have almost continuous pain in my joints…after 3 or 4 seconds, it completely disappeared”, or MS's aches and pains which “go away completely… You're not in this world, you're in this other world…[where] you're sort of pain-free, *everything*-free, aren't you!?”, up to the unadulterated body-bliss of “ecstasy” (4). BW, here, embodies more of a “post-orgasmic” (1) aftermath:

“Like ^*^eeeeh-boosh!!^*^. And then all that jerkiness afterwards was like the comedown from having just ejaculated… It felt sexual in as much as it feels like it peaked, then post- now I'm just kind of this dribbling, jelly-like twitching thing”.

#### Neutral or both

Notably, eight cases (22%) mentioned body experiences of a more *neutral* type (either pleasurable or uncomfortable). The spontaneous making of “religious hand signs” (3) such as “instinctive mudras” (JA), or “dancing, moving with this shape, and I notice I like to do [these signs] when I'm in this space, to [the entities], with them. And it does remind me of sort of Hindu mudras” (RH, trip 2).

In both TM's first and second time, he convinced himself of having “convulsions” (3), purely subjectively, as he “was seeing myself here on the sofa…I had seizures for like 10 minutes in my trip!”, or other contortions which he reconstructed in the interview “even more than this, like *The Exorcism*, like bent over… my body reacted…very violently, and I wanted to apologise”.

AZ, sitting in his garden, was the sole participant to feel himself transform, in his case, into plant life in the act of apparent “phytanthropy” (1):

“Then all of a sudden I could see the entirety of myself placed as the heart of a tree… I could see branches coming out of my torso. And on each branch, there were beings that resembled a bottle with legs, and they were marching back and forth infinitely. Weirdly enough, I could feel all of their movements inside of me”.

### Sensorial: A kaleidoscopic blossoming and blending of the senses

The sensorial domain of experience is drastically transfigured in the psychedelic DMT state, but this category is concerned with the predominantly visionary experiential components.

#### Open eye

*Open-eye* visuals, noted by 11 participants (31%), were either prior to their immersion into a separate space (see Michael et al., 2021), or when fleetingly opening their eyes. A further “reality breakdown” (3) was witnessed—sometimes a “pixilation”, as in TM's (trip 2) case where the researchers “were very pixelated, with big pixels…you were like a very old video game from [sic]70s. *Worse* than that!” MS both saw her fairy-lights “morphing into…loads of skulls” and gasped that

“Oh my god, there was like a flow! Freya [MS' dog] was moving, she was like the energy flow between me and [DMT partner]!... vividly moving…energy between us all. Because everything breaks down- you all break down into something or another”.

Another particularly eye-opening impression communicated by GR was very “clairvoyant-like” (2), in the sense of wielding an experiential prowess uninhibited by the regular senses:

“each individual atom of that particular information which I'd received from the outside world, was just like – you could go into it as much as you want, or go out of it as much as you want… every pixel of everything was more like a tunnel…and I could choose to go through it, and just choose where to go. It was like a complete[sic] freedom in that way…

So this [chest of drawers] was actually…transported into a huge field of minute atoms or something. But I could go in it or outside it… this [carpet] was more like I could see through it, so like instead of it being a thin layer fabric it was more like something huge and I could go and see each detail of it”.

#### Visual

Changes in the *visual* domain (eyes closed) were offered by 27 interviewees (75%). This was the fifth most commonly reported super-ordinate theme, largely in the colorful kinetic geometric-fractal dimension (virtually quintessential in the literature), with several of our DMT visionaries invoking a “kaleidoscope.” The “geometry” (16) often defied description whatsoever, DS matter-of-factly retorting after being asked if he could describe them, “Not a chance.” Similarly, BB blithely comments on “their intricacy and the fact they're infinite and all that”, and MS stresses that “it's a busy other world and shapes and fractals and just absolutely mind-boggling!” More than the enrapturing aesthetics of these forms, their being of a sentient quality and united nature with oneself (see Michael et al., 2021) is succinctly accentuated by RV:

“I was transported into precisely the place that ayahuasca took me on the previous journey. A place of unbelievable hyperintelligent geometry and shifting forms… it's so, so difficult to describe. But it's colourful, it's everywhere, it's moving, it's very, very precise, it's very form-full – and it's *you*, you know! You kind of realise that, whatever it is, *you* are too”.

This remarkable initial display is often the membrane penetration through which affords the experient their “other-dimensional” venture, as in JA's case:

“It's almost like you're being pushed through this tiny gap in the geometry that you have to sort of go through to come through to the other side. Then I was sort of twisting and going into it and coming through it, and then I knew – OK, I'm here now”.

The geometrics were prominent in the “fractal” design (11). AZ, again highlighting their sensible or interactive properties, informs that he “could somehow manipulate them and feel them…all I could see was these strange patterns of sacred geometrical figures which were infinitely drawing more geometrical patterns out of themselves”. RH (trip 1) sketches the potentially overwhelming saturation of this multiplicative effect:

“everything was going back that way, down up. And not just that, every single part of it was fractalizing, there was nothing to hold onto, nothing I could grip, or do anything. Everything was just expanding from every point, no end to it”.

The significant kinetic energy of both the geometric-fractals and the other world at large, i.e., a state of “flux” (13), may also often preface a suddenly more stable or static other world. This is pithily gestured to by BB, expressing, “it was just crazy crazy crazy visuals – BANG, this thing, and just this thing.” Notwithstanding, many others' entire scenes continued to be charged with this dynamic nature where “there were a million things happening, switching from one to another” (GR), such as JM's flowing tapestry of ever-gyrating cog-like structures, FF's teleportation into a wildly bustling biomechanism, and the dynamism of EM's fluid machine-like construction—all resonant with the mechanoid *and* organic imagery (see Michael et al., 2021). One explication of this effect was given a poignant spin by MS around her vibrant, fractal skeletal motifs:

“That's what it taught me, the impermanence of everything. And everything's moving, nothing stays still. Nothing. That's what it really, really tells me a lot…and the sooner we realise it in this reality, the better the place we'll be

Interviewer: …you've also got all this mortality imagery as well, so is it like our lives are transitory?

It's exactly that, exactly what it is. We're only here for a blip, and death isn't a bad thing”.

Again, implicit in the ineffability of some of the patterns seen (and often in the more iconic visions) is their occasional “hyperdimensionality” (8). RS riddles us that his “very intricate…dancing lattices” were “at least 4-5 spatial dimensions… sheets blowing in the wind – but hyperdimensional bedsheets!” GR describes a mind-bending folding of his awareness through impossible angles, and RH (trip 1) witnesses a garden that was “so much more than anything 3D”, where such emphatically peculiar spreading of space is valiantly rendered in words by both SH and JM:

“one bit where it kind of turned like ^*^Voowwp^*^, and went kind of angular, a bit ketaminey when you kind of go, ^*^Zzzz^*^ and you shift into weird dimensions, or things change size or you become a different format…it was almost like I had become small and underneath a piece of furniture or something? This is *not* what it was like but the best I can try to describe it” (SH).

“All this spinning, all this disorganised space that you can't quantify in 3D space… It's not necessarily linear… things spinning[sic] in multiple layers of things. It's very hard to describe in space terms… everything made sense wherever it was, even though it wouldn't make sense in a normal situation” (JM).

#### Cross-modal and other

Further to the purely visual, *cross-modal and other* sensory features were conveyed by 14 experiments (39%). “Sounds” appearing during the experience vs. at the onset did not seem very common (4). MS describes something of a more continuous sound, “^*^Rauuuughh^*^ – there's this sound!... a sort of sound that came with it, like ^*^Rrrrrrnngg^*^? Weird… A frequency vibration sound… every DMT trip I've ever done it comes with it”. ST adds to his “Borg cube imagery” that it also possessed “accompanying synaesthetic glitchy sounds”:

“The hypercube was like a placeholder for the many such images I've…seen projected by [Video-Jockeys] to accompany tracks. The same quality of glitching that…populates psytrance DJ sets… there would be this coupling of the sound to the image and then my brain, my mind would kind of follow it and be like ‘yes yes yes!”'

ST here introduces “synaesthesia” (10), appearing to be experienced by a significant number. Here, it is of a visual-auditory type, while next, he seems to describe a somatic-visual complex with cognitive-affective components, a hypercube vision corresponding to his experience of difficulty breathing and, indeed, to his self:

“It's almost like I'm seeing what I'm feeling… it was that same process of teaching me to associate the feeling of…this ‘autobots cyborg’ thing, that I could visualise as my experience of Me… it's almost like watching yourself being sliced into a billion different pieces…

Seems to be more like a multidimensional movement going on, different type[sic] of components that all appear to still be connected doing this kind of movement… it seemed to be a process where it's saying, ‘Imagine this represents you’, and then that realm, that imagination immediately makes it represent me, such that whatever is happening to *it* I am feeling, or rather is transmitted to whatever part of my experience”.

Of note, ZD in addition to disclosing some dynamic relationship of her breath feeding into the geometric visions—in turn also associated with her sense of self:

“I became really conscious of my breath…I can almost see it… very aware of the breath as all this geometric stuff starts happening and it's like the breath is *visualised* as a pillar in the centre of it all, and it might be like…a white thin line in the centre of it all, and it's expanding and contracting as I'm breathing, and then it sort of blips into nothing – and then that's where *I* disappear”.

### Psychological: In inarticulable loosening of the psyche

#### Memory and language

As per the cognitive repercussions of the breakthrough, faculties of *memory and language* were impacted dramatically, as exclaimed by a full 31 participants (86%). As strewn through all the above, very many found their journeys so out of this world as to be “*really* hard to put it into words”, where they usually mandate some “recourse to metaphor” (19), and as evident throughout similes are also heavily relied upon by subjects. Though, even this is not without caveats, as ZD exemplifies that “it's just so subtle, that if I use a certain word it will be really misleading”, DS fails to express “this thing here in the middle, whatever the fuck it was, I wanna say it was a serpent but it wasn't a serpent in any way”. SH's words about her “beautiful patterns” let her down as they were “all, ^*^Vvrrr^*^, coming around these rooms- they *weren't rooms*, but rooms”. When asked to attempt the medium of drawing instead, RH (trip 2) admits, “I will, but I'm concerned that this human mind would distort [the entity]” which he witnessed as being “a bit octopusy[sic], but that would just make it very- you can almost forget that”, as well as JB's retort that his trips are “more a feeling, sort of sensations. I just feel if I try to draw, I'd be rationalising it and the wrong side of my brain's gonna be trying…” JM sympathizes too, where ephemerality of the memory of the experience impedes its capturing: “It's really hard to memorise, isn't it? It's just so abstract, you're clutching at things that you thought you saw!”

“Temporary memory loss” (15), that is *within* (vs. after) the experience, pertains, for instance, to subjects' suspension of their short-term memory, like the knowledge of being in a psychedelic experiment, especially at the trip peak (minutes 1–2). EM, for one, attempted to “'keep the question in mind and remember that you're a research subject' – but I kind of didn't after a split second”. Many others resonate with this, but importantly too, alluding to a profound interruption of their selfhood, a diminishment in one's longer-term autobiographic memory (similar to an “ego-death” effect, yet without an explicit *loss* of the individual perspective, and also without concomitant mystical features), such as SP poetically painting the picture of becoming a blank canvas, where he “just came to…with the feeling of, it sort of becoming from nothing. There wasn't much in between. Nothing. Like waking up – without any dreams”. FF also thought provokingly recounts that “I wasn't really aware that I *didn't* know who I was… kind of like ‘Wow, here we are, let's just observe and swim and just take it!”’

This occluding of one's prior conceptions of personhood and world is reinforced by RH (trip 3), convincing himself even in his trip that “there's no way I'm gonna even remember this, I'm completely in another world, with only a tiny remembrance there's a human world going on as well”. Therefore, GR evocatively explains that

“I didn't even know if you were real or not, or is this real life or not, or reality or not… There was no reference point, no nothing… what would have brought me to have seen more to it would've been…if I were to keep my eyes closed – but I didn't have a conception of eyes, so it's very hard…

So I remember thinking, ‘Where am I!? Is this going to end? When is it going to end?’… Because I lost my memory, or what I did, or the fact I took DMT – that did *not* exist, that information was not there anymore... As if I'm just like teleported without anything from the past…into a place, ^*^Peeww^*^, just like, ‘What the fuck!?’ – I mean, I still had my language capacity!”

Accounts of “looping” were given on a few occasions (4), such as BW's complex replaying mix of mental imagery (which was also highly synaesthetic):

“it was so weird that it was…like an OCD-type intrusive thought that kept popping into my head… almost as if I was still talking to you like I was sober… the intrusive thought was a recent memory of a recent episodic thing…

The loop kept bouncing in. It was like ‘what the fuck was that’, and ‘did you all see that!?’ And then it's like…did I actually say that? Then it went, then it popped back in and then… ‘Did you all see it as well?’ Then it's like, but what *is* that!?... Then it's like, I've actually lost my mind, I've lost the power to know the difference between talking and thinking…

that really weird feeling of transparency, almost like a schizophrenia-type, am I saying it or thinking it…? …And then this sort of realisation that actually, I'm probably not talking at all, and this is all in my head… ‘They're gonna think I'm crazy”’.

#### Awareness and sense of self

The most salient alterations within *awareness and sense of self*, noted in 18 cases (50%), involved more infrequent retention of “lucidity” (5)—the sense of “preservation of ego” or sense of self (as contrasted to the above, more common, loss of self-related autobiographical memory). AB emphasizes that “I was always aware that I was me and that I was having a DMT experience…but reality had completely been replaced”. LG, although he was “dead in an icicle” and his “[etheric self] went into another plane… I was still me – my thought processes still felt the same… slowly I was like ‘I *am* dead’, but my ego was still here, so death is like…not that big a deal!” This staying within selfhood is sharply juxtaposed to the dissolution of the ego, the latter explicitly verbalized to a greater extent (see Michael et al., 2023, SSRN, regarding the mystical experience). An ambiguity, however, or even fluctuation between these states is suggested by JB:

“I was quite kind of lucidly half there, half here at points… Didn't have any sort of- feel like I met any external entities – other than a Oneness, an Everything entity, a Universal entity…God, or whatever… I was kind of *in* it...

Interviewer: But did you have a sense of you[sic] own identity still?

That's what I kept on laughing at myself that I did – in a way… I felt totally [unitive]… there was a certain self-awareness still. I don't think I completely lost myself”.

### Emotional: A soaring angelic, and fathoming hell, of the soul

#### Positive

DMT incites a profoundly emotionally pervasive experience. As viewed by 34 cases (94%), that is, almost *all* the experients—and despite the somewhat frightful experience onset—it becomes an overwhelmingly *positive* one. The trip was most often noted to be simply “very pleasant, very calm” (16), “really safe…so secure” and “so harmonious”, for instance for EM it was “such a smooth trip, so nice, so lovely” to be amongst her “community of harlequins”. TM (trip 3), admitting that though he “was coming from that torment in the beginning” suddenly “it was very peaceful… I was almost expecting to hear birds singing!” This “bumpy ride” at the onset was replaced by a more tranquil, “friendly kind of visit” is shared by numerous participants, such as JM:

“if you're not getting a bit anxious when your head's falling about, there's something wrong with you – but beyond that rush at the start, it was friendly… very comfortable, [sic]little bit warm and fluffy… I was smiling”.

The welling of “loving” feelings from the participants and experience of “connection” with others (10) was oftentimes deeply engendered. This may be associated with the mutual love generated between experient and entity, such as RH's (trip 3) uttering, still communing with his entities, “Oh I love you so much, it's like we are one thing. Oh, I love it here so much!”. In BB's vision, he encountered representations of his wife and son, which struck in him an epiphany of the essence of the agape love form:

“the feeling…of intimate connectedness with other people which I crave – desperately… It's the kind of feeling that encompasses one type of love which is in all the participants equally. So it's not as if, he's a kid who feels one way about me, and I feel a different way. No, it's just one shared form of love…crystallised into this kid figure”.

The following two evoke an engagement with greater consciousness, from whom they could not wholly unembed themselves (echoing the dynamics of selfhood above)—their reflecting on which caused tears to stream from their eyes:

“Wow. Just love. I don't know what to bring back from that. Woah… it was not so visual…not so cerebral, just this utter sort of all-bodied sort of connection… and just reminding myself, that ‘I asked for this’, and that I love you all, and that I love everything, and fucking- How much trust and love- [begins to cry, and laugh]…

I…shed some tears, but tears of beautiful- very happy tears, laughy tears. I kept laughing at myself for thinking about myself as ‘I’. My mind would say something like, ‘I love it, I love everyone’. I was like ‘I’m still there, I’m still saying “*I”* love everything, I'm still stuck on this *I”*!” (JB).

Though there is less levity, RV's pathos-filled account was also hued with striving to embrace letting go of the self amongst his similarly heart-opening encounter, here referring to the loving life to which he aspires:

“It would be something much more fluid, about a much more spontaneous expression of love on a day-to-day basis. It would be about hugs, and about blessings, and giving, about never hoarding, never requiring[sic] [begins to cry]… I have actually thought about being a priest…someone whose job is blessing and love and giving and modesty and gentleness and slowness [crying, hugging interviewer]… That is why we're *here* isn't it?...

Yeah, and there's this feeling that if I release my heart-centre, then my life has to change… to be…some kind of vehicle for love. I feel like I can be, but I've just resisted it my whole life, you know…

Interviewer: …You've got a big heart [RV]!

Yeah, but it's what we do though to shut it down”.

The term “beautiful” is one of the commonest positive descriptors thus far volunteered, sometimes in connection to the aesthetics—but more deeply evoked by the encounter or mystical-type experiences, where the co-resonant “profundity” (14) captured many experiences. FF, despite going in with the intention of “sorting my bloody head out” and ending up as “just a spectator of this alien phenomenal environment”, resolves that it was one he:

“felt privileged to be seeing… I'm blissed. I feel *blessed* that I've had this opportunity to go there… what's lovely with these things, is the profoundness of it is in itself an insight, and then that…leads to maybe helping with the intention…if you work on yourself”.

JB resonates with such otherworldly beauty and profundity being fundamental in its own right despite not necessarily receiving immediate, practical answers:

“I was in another world… Yeah, it was full on, beautiful. Just totally blissful… But it leaves me wondering like, ‘What else?’ Or kind of, ‘What do we do with that?!’ Yeah, but in itself, it's just a powerful experience. To remind me of the abstract nature of reality, made me kind of feel this different universal plane. Which[sic] just feels really profound, and really beautiful to remember”.

Basking in such supreme profoundness on occasion elicited feelings of great “humility” (3). RV, here, manifestly wrestles with the ultimate truth of the accessed realms in stark contrast to the egoic self, which in turn is sometimes not strong enough to incorporate such truths upon returning:

“And it was so astonishing and so humbling! And it feels so ridiculous in a way because there you are [makes squirming noises] just a tiny struggling ego… anything that's truthful for me has to connect to the truth…of this reality of what's presented [in the trip], and you know, these dimensions beyond waking consciousness…and all their manifest healing… And really anything less than that- the fact is my life *is less* than that… as yet I've not made my life about bringing that truth to humanity. I resist *that*, and that's what the last ayahuasca journey was about as well, was to stop resisting *that;* this *is* of ultimate importance. This is of ultimate importance – but it's *so* disorientating”.

Eloquently further parsing out his “cosmic giggle” (see *Cosmic game*, Michael et al., 2021) and mirroring FF/BB's sentiments of welcome unexpectedness, BB frames it by a particular way the ego can be put in its place by DMT:

“Another reason for the giggles was…I said to you I go in usually with a question and it never gives you the answer to that question, it gives you the answer to a question you *should* have been asking. It's just, a kind of deflation, of what's left of your ego, just to say – ‘yeah, you really didn't have a fucking clue, did you!’ Kind of this feeling of ‘ok, alright like, I give up, whatever, cool, great – what else do we have, oh awesome, clouds!’... the greedy thing you go in with – it says, ‘you didn't need that, SMACK, dickhead, you needed this! So go on and enjoy’… So that laughing at yourself[sic]”.

An experience of “healing” (5) within and by the DMT state in itself was demonstrated by BB again, as part of this ego challenge here, and surprise revelation of love and lack therein (as at the start of this section):

“there's a particular type…of emotional closeness, of love, basically, which I'm constantly searching for…in particular for my son – fully opening up to that, which I feel like I do. But I just realized, I probably *don't* completely. But…the intensity of that feeling, that's the medicine that I need. Just this feeling that there's like *another* layer, another type of love…

there's this other thing, always in the background of my mind – ‘Why do I feel I'm not loved enough?’ And there was something, some little lock somewhere inside me, that I haven't accessed… Just this sense of, you know: take 5% fucked up your whole life, and if it was because of this thing, and all of a sudden – ‘that's what it is you idiot!’ And it's really acute and obvious!”

JA also required a suspension of ego to facilitate deep spiritual healing, coupled with shamanic-like ritual purgation:

“So if I can connect to whether it's a sense of fear, or sense of an element of lack of self-love, and then if I can make the right sound, the right frequency, that will help to purge that energy which is sort of stuck within me…

I realise when I'm in that state of consciousness how much I carry on a daily basis [in terms] of difficult energy, blocked emotions I'm not able to pass by or release. And…it just feels like we're in this pure divine state where we can remember how to process the energies that are difficult to release in everyday life”.

This purgative energy is very akin to the intense emotional “release” or “relief” (4) experienced by some, such as SH and AF's highly resonant encounters imparting insight into the *Cosmic game* (see Michael et al., 2021). In AF's case (as with RV), this power was reminiscent of ayahuasca:

“the second part when I laid down…and the crying, it felt like the ayahuasca… I'm just comparing the two things, because it was very liberating, and just like… Yeah, just *be*! I had that sensation of just existing. And I want it to last as long as possible… I just opened myself… Wow, this is powerful what you study [laughing]. My goodness… even the crying was like ‘Yeahhh’, crying, just ‘Ahh, let it go”’.

SH shares this impression of simple “isness” accompanied by such liberation:

“I was like This is The Game, you just go, step into experiences, and see where it leads you, without meaning to have an expectation or lesson or story or reason. Just what is just is. Quite profound… Amongst the silliness – it's so liberating. Often when I get these downloads from trips it feels quite heavy like ‘Arggh’, this just felt like ^*^Pffwahh^*^. Amazing, like a big release”.

While these feelings of profundity, humility, and healing can be of serious substance, these are time and again intimately wed with experiences of “humour” or even “hilarity” (7). This is well-embodied by this above gamification of the cosmos, received by SH as she juxtaposes the profundity and silliness, which catalyzed eruptions of laughter—as well as BB's celebratory giggling earlier, being granted the medicine he did not even know he needed. Not dissimilarly for JB, the humor hailed from trying to reconcile identifying with greater consciousness:

“also a bit of chatter about ego, and ‘*not being special’*. Because part of it is, there is a sort of, ‘Jesus’ sort of feel, an enlightened ego thing. You feel like, ‘Wow, I'm so special!’… Then this kind of laughing realisation about what you're saying and thinking, and how absurd it sounds to yourself!”

Understandably, this volume of emotions of such depth and positive valence incited in many subjects a “gratitude” (8)—from MP or RH (trip 3) repeatedly uttering “thank you” *in situ* as they tripped to MP explaining that it was “Just the opportunity, and the healing, and just being alive to witness it”. AF responded, in regard to her above freedom from her mind, that she's “bloody grateful” to be able to “enjoy all the things that we forget we are”—but also her humbled appreciation for being gifted the experience, either of the DMT or her own life, asking herself, “Is actually all this time dedicated to me? Like I always had this feeling, it seems all for *me*. This is where the crying comes from [begins crying].”

The thankfulness of OR (trip 1), who belongs to an Afro-Brazilian syncretic ayahuasca-using church, was less for soaring angelic, but for averting a fathoming of hell, explaining that, upon having just witnessed the struggle endured by his non-religious DMT partner:

“in my heart, as I may say this, I was very thankful for having a doctrine or a religion in which I go. Because I was literally hearing like, 'You know what happens when you're not prepared for some stuff'. And the spirit guide was like, ‘You're cool! You have this [doctrine], it's OK!”’

Several experients expressed their appreciation for their entities, for the teaching and healing they bestow, such as RV, who

“felt them trying to work, even in that short space. As the entoptics faded slightly, I got the impression, the sense of…several entities starting to try to work on the tension in my jaw… they saw that some of the trauma is locked… And I was grateful for that”.

An impression of “familiarity” was oftentimes felt around the beings visited, but frequently a familiarity of a certain scent, a “déjà vu”, pervaded the experiential core (11), as gestured at by JB's framing his “different universal plane” as like a return; “to remember” after having “gone back somewhere”. Resounding this anamnestic process, RS shares, “I always feel very comfortable there, often I feel like ‘Oh yeah this is where I was before, and this is where I'll go after, I forgot about that!’ sort of – ‘Cool!’ [laughs] Like this lifting of this amnesia”. The conviction can be carved deeply in participants' minds, as conveyed by AF, “I know I was there, it's again like all the time, I know this place, its familiar. I've been there before, I know, I know, I've been there before!” Yet again, this feeling breeding ideas of origins is motioned by AN:

“It felt like I came from it, I'd come from that submersion, that energy field that I was in, it felt like that was where I come from, so it felt natural to reassemble myself from that space. It felt familiar…doing that reassembling[sic] myself, felt natural. Something I'd done before”.

As tacit in these accounts (and reverberating with descriptions of the familiar beings, Michael et al., 2021), this unassailable recognition is one which runs beyond partaking in DMT on prior occasions, as clearly substantiated by FF, referring to an immersion into some natural, yet beguiling mechanism:

“the intensity and the place you are, it's so phenomenally alien, but familiar as well, but shocking… That second I went in and that sense of fear, like ‘OK I've been here before’…

Interviewer: And do you think it's familiar over and above the fact you've been in this place before when you've literally done DMT?

Oh yes, oh definitely! No, I remember thinking this the very first time I did DMT, it's somewhere deep down…that it's a friend, you know… it's really interesting how it is such a familiar world”.

#### Neither or both

Many other parts of the experiences were not patently positive nor expressly challenging, but *Neither or Both*, as described by 21 (61%). This predominantly comprised statements as per the “intensity” of the DMT state (21), largely felt to such degrees as becoming “overwhelming”. In reference to requesting an intensity rating out of 10 for the whole trip, it was more often than not heard that “it was 10. Yeah, there was nowhere else to go”. Often in the experience's initial stages, interviewees were adamant “it had gone right off the scale” (RH 2); “It was 11 – it was fucking mind-blowing” (RH 1), “it just seemed hilarious, because it was so fucking intense that I could only *just* understand the question” (ZD), or in MS's case, “it was so, so powerful all I wanted to do was make sure I was breathing”, left only to “hang on for dear life!”. This echoes the psychical deluge, the “submersion” discussed at trip onset, where finally, for several, the overwhelm transpired amidst the throes of the collapse of their personal world, such as RH's second experience within the study, which “felt like death” and “was so intense…that the ego fractured”—or like during GR's efforts to ground himself:

“It was very overwhelming... And I remember I was just going, my sense of self dissipated, I just did not exist anymore… I opened my eyes at some point, and that was my baseline reality, which was quite comforting in a way because I was looking for an anchor… there was just so much going on, like what the *hell* is going on!?”

The ability to “let go” or enact a “detachment” (3) despite the initial buffeting received was manifest in OR's (trip 2) adept approach, learned from his religious practice:

“It's like a sense, a feeling I shouldn't just trip, I should understand this is a tool; I can just go with the flow, or I can- it's a different perspective from the neurotic position like I *have* to control this – it's like ‘Eh, I can be in the sea in the waves and let them throw me on the rocks, or I can learn how to surf’! It's the same space and place, but my perspective on that space is different. I can just be [makes drowning noise], or [makes flying noise]”.

#### Challenging

Experiences which were admittedly more *Challenging* than this occurred, but to a substantially lesser degree, divulged by seven subjects (17%)—and of these, perhaps only three journeys were marked by this on the whole. This maybe particularly so for RV, whose ego's “fear of letting go” (1), juxtaposed to OR above, has already been touched on in his awe-struck encounter and desire to embrace selfless love, which he here develops in even more profound depth around “re-experiencing past trauma” (1), also emotively catapulting him back to prior ayahuasca work:

“so my ego is not sufficiently developed to let me go into that, sort of to allow the self to disappear, to allow the swallowing of the self into that ‘Super-place’… so it uses trauma or at least a kind of foundational fear that…I know…exists in childhood- fear as a way of keeping a tendril, preventing the swallowing… so what I see in that space is that this fear I have which stems from this trauma is, well, like a way of keeping me separate…it's a way of preventing that ecstatic self-losing…

letting go and moving through this experience requires this very special, precious loving non-attachment… Which[sic] means a certain letting go of your nearest and dearest as well… in the last ayahuasca journey that was a big challenge for me; 'In order to experience this, you have to let go of [your wife and daughter]'. That's what they were telling me- Are you telling me I have to literally leave them!? It wasn't that, it was in order for you to excise this, you have to excise what is essentially a fear, a fear of loss isn't it? It's fucking hard”.

Not unlike this experience of RV refusing to accept saying goodbye to his beloved—a stunningly reminiscent type of “grief” (1), yet one from being forced to leave loved ones behind after having “died” *oneself* , as well as the resultant “guilt”, was characteristic of LG's uniquely chthonic journey:

“I was like, so I'm dead, I *am* dead now. Then I was looking around in this plane… I was…viewing my face, like the[sic] shellshock, the magnitude of what's just happened. There was almost like a feeling of, I guess it was grief that set in-[sic] grief, but not for me, but for *her* [LG's romantic partner]…

I was like ^*^vrroohhmm^*^ into this ethereal aura of whatever icicle thing. Then it was just trying to cope, and acceptance of the fact that this thing, this love is ended, and…she's gonna have to deal with the grief and pain…

I was just there like ‘What the fuck is this!?’, I couldn't even pay attention to the surroundings that much because I was so distressed by the fact that I was dead [laughter]!”

A further and final category, “Meta-narratives: Co-creative Insight into Heart and Cosmos,” acts as an addendum to the above analysis, where an unabridged description is provided in the Supplementary material 1. This is owing to the content of answers not deriving directly from questions within the SSI but independently volunteered. Mainly, they are not explicitly pertaining to the experiential content in itself, but rather more interpretative comments upon the experience, and as such, not easily comparable with Michael et al. (2023, SSRN) comparison with the near-death experience content. The reader is encouraged to refer to this in Supplementary material 1, given the nature of responses surrounding emotional breakthrough (Roseman et al., 2019; Davis et al., 2021; Timmermann et al., 2022) and ontological belief (e.g., Davis et al., 2020a; Watts and Luoma, 2020; Timmermann et al., 2021, 2022; Nayak and Griffiths, 2022), relevant to many recent reports.

## Discussion

Overall, the present thorough thematic and content analysis has illuminated the DMT state as encompassing the *onset*, though often difficult labor, to then giving birth to a profound DMT breakthrough, which presented an all-pervasive spectrum of experiences. These experiences spread across all of the *bodily* domain, marked mostly by *dis*embodiment, the *sensorial*, being predominantly extravagantly visual; the *psychological*, transforming the core of the sense of self; and the *emotional* domain, which was almost universally desirable. This, therefore, is demonstrative of the drug's capacity to take one out of themselves and to engage with another, normally welcome, reconfiguration of all areas of one's own consciousness.

### Comparison with other DMT studies

Representing the one other published thematic analysis on the DMT experience, Cott and Rock (2008) coded 19 reports from an online survey, resulting in nine general themes. These entailed—distortion in time, space, and self; hallucinations (visual, auditory, or bodily); veridical hallucinations (i.e., considered to be true or real, including sentient beings, see Michael et al., 2021); affective distortion: involving intense euphoria and anxiety, typically during the “DMT rush”—relating to *emotions, pleasurable or challenging*, in the above analysis, including at *onset*; spirituality: including *beauty* or *love*, relating to the same themes in the above analysis (also see Michael et al., 2023, SSRN, regarding the near-death experience). Finally, directly linking with themes herein, there is familiarity; lucidity: describing an ability to “fully appreciate the experience as if in an ordinary waking state”; ineffability: denoting difficulty capturing a non-linear, nondual experience; extreme intensity: where participants became “besieged by DMT-induced cognitions”, often leading to anxiety.

Timmermann (2017) delivered a preliminary overview of the prominent effects of 7–20 mg of injected DMT among 13 healthy subjects. These encompassed themes pertinent to Michael, Luke and Robinson (2021) analysis, namely complex imagery (e.g., which was familiar to 62% of participants)*; a* sense of presence (e.g., showing intent by 69% of participants); a sense of receiving information (46% of participants), and sense of being transported (e.g., to otherworldly space, 69% of participants, reminiscent of the current study's *Space-time shifts*). Themes correspondent to the present analysis included *bodily* effects (100% vs., in the present study, 28% *pleasurable*, 22% *neither*, 25% *uncomfortable*) such as an onset including a rush (vibration and warmth), distortion or dissolution (see “disembodiment”, 53%, in Michael et al., 2023, SSRN), and pleasure; simple visual imagery (100%) which were *geometric* (100 vs. 44–17%), *colorful* (77 vs. 36%), *kinetic* (62 vs. 36%), including “vortex…forms”, which may be “cartoonish” or “exquisite…resolution”; and finally emotions (92%), which were predominantly positive like humility, love, gratitude, or peace—where spiritual feelings and inspiration may correlate to this study's “profundity/beauty”, and acceptance and openness, may correlate to “loving/connected”. Anxiety/fear (31%) was also coded, corresponding to this report's range from “terror” (at onset, 17%) to “anxiety” (during the experience, 8%).

### Comparison with other exceptional human experiences

When comparing this study's themes characterizing the DMT space with exceptional human experiences (EHEs) of other kinds, as stressed in Michael et al. (2021) study, “alien abduction” or other alien-encounter experiences (Mack, 1994, 2000; Hancock, 2005) may be particularly evocative of the DMT trip. Some subjects' feelings of being “trapped” are similar to the sleep paralysis experience, compared to the alien abduction experience (mainly its first phases; Blackmore and Cox, 2000). The often deeply *pleasurable* states, under bodily experiences, are resonant with the sometimes ecstatic episodes reported by abductees, one asserting that she would “gladly sacrifice her child” for that feeling (Mack, 2011)—and the “vibratory” states here are also classically reported by them (Mack, 2000; as well as in OBEs, Montenegro, 2015). The *visual* experience, under the sensorial category, of the “organic-mechanic” quality links also to Luna (2022) and Kripal's (2022) attestations that, in the former, the witness leading to the coinage of “flying saucer” described the structures as living beings, and in the latter, the serpentine mother-being of ayahuasca is sometimes experienced in the form of a UFO. The emotions of a *positive* nature, such as “connection, humour, and healing”, are finally congruent, respectively, with abductees' frequent reports of feelings of oneness, including with the ET beings experienced (Mack, 2000), these entities' trickster-like characters (Keel, 1970), and their spontaneous healings, emotional and *physical* (Strieber and Kripal, 2016), with this latter particularly akin to *RV*'s healing (the beings also described by him were “mantis-like”).

Resonances between the shamanic journey and DMT are also obvious, where such DMT-containing “entheogens” like ayahuasca, or the snuff *yopo*, are employed by shamanistic cultures for divinatory and healing purposes. The descriptions of this study's participants of “submergence” at onset are arguably gesturing toward, especially with its watery dimension, the descent into the underworld (Hillman, 1979), but also the evident struggle of the self, and “competing currents”, suggestive of the shamanic experience of disintegration (Narby, 2022). Not dissimilarly, the *emotional/body-*related feelings at the beginning of fearing for the integrity of one's life echo this shamanistic rebirth motif, especially in light of propositions that historical near-death experiences acted as the basis for the development of shamanic societies' ritualistic technologies to replicate this experience of dying (Shushan, 2009, 2018). DMT's “organic-*mechanic”* aesthetic highlights the particularly entheogen-utilizing shamans' entering into (UFO-resembling) shining, metallic structures (Hancock, 2005).

The near-death experience, finally, has evidential overtones of the DMT themes herein. However, the precision of equivalence between the states may have been thus far over-estimated in the literature (see Michael et al., 2021), and this question is the dedicated purpose of a subsequent report (Michael et al., 2023, SSRN). An abridged version of the similarities (vs. differences) between the analyses herein and the NDE would include sounds at onset, sensations of dying, sentient light forms, preservation (most NDEs) or a diminishing (minority of NDEs) of the sense of ego, and feelings of peace and love. One example of a lesser discussed NDE feature echoing DMT is the present themes of “hyperdimensionality” (also reported in Strassman, 2001) and “clairvoyant-like perception”, which are reminiscent of the so-called “omnidirectionality” of the visual experience of some near-death experiences, such as in the initial OBE (Ring and Cooper, 2008).

### Putative neural mechanisms

#### Dream-like activity

Closed-eye DMT administration has been found to reverse cortical traveling waves from backward flowing (carrying top-down predictions) to forward (carrying bottom-up errors/sensory data). This is consistent with a reduction in the precision weighting of “priors”, i.e., predictions made about experiential causes and together constituting an internal generative world model, which suggests the brain's action as if it were receiving novel visual input (Alamia et al., 2020). The key other natural endogenous state sharing much with the DMT trip as delineated in the present study, including such complex visionary states, as well as intense, broad emotionality and perturbations in self—especially interactions with others in intrinsic sensorial worlds—is that of dreams. Dreams are the prototype for “disconnected consciousness” in which internal awareness is present despite apparent unresponsiveness—where the major neural correlates of the dream-state appear to be shared with the psychedelic and dissociative DMT state, that is, suppressed alpha cortical oscillations and elevated theta and delta (slow wave; Carhart-Harris, 2007). Both were correlated with the intensity of visual imagery under DMT (Timmermann et al., 2019)—with the theta–delta being shown in provisional analyses to be over the temporal association cortices, as in dreams (Timmermann, 2019). The theta–delta enhancement is also inversely correlated with the (more non-dual) mystical experience (Pallavicini et al., 2020), substantiating its association with the dualistic interactive style of the DMT breakthrough. As such, the DMT condition is akin to a lucid dream, albeit very unique in that the brain-state may be REM sleep-like, yet the individual is technically wakeful and typically experiences the state as real. The TPJ, possessing a key component of the DMN (the iPL), is also pivotally involved in the dream state (Scarpelli et al., 2019), where the release of the DMN's inhibitive effects on temporal lobe structures (Carhart-Harris et al., 2014) could also stimulate this region—and elevated global connectivity within the TPJ and insula has been reported at least under LSD (Tagliazucchi et al., 2016).

Relevantly, such DMT and dream theta and delta activity (also increased in other, for instance, regressed near-death states) is linked to the temporal organization of episodic, i.e., sensorially rich memories (Buzsáki and Moser, 2013; Martial et al., 2019). However, such episodic memories are typically autobiographic—which may be re-organized and reimagined in REM sleep, but the personally recollective nature of the DMT content is not self-evident. Indeed, much DMT phenomenology can be considered to bear little resemblance to that of dreams, suggesting important neural divergences also in their origins.

#### The entity encounter

Many participants in the present analysis articulated experiences in reference to entities, such as the sense of unity and familiarity with them, love felt for and from them, or healing received. As discussed by Michael et al. (2021), top-down cognitive mechanisms conferred onto basic sensory data may construct social imagery, where computer modeling of this effect has generated examples of anthropomorphic visual forms (Aqil, 2022). Dissociative identity disorder (DID; formerly multiple personality disorder) may also be instructive, a condition in which “alters”, secondary personalities may be considered projected “hallucinations” of the fragmented self (van Heugten-van der Kloet and Lynn, 2020). DID has similarly been compared to the dream state, with in-dream relationships being a prototype for DID, but which also may be precursive to the disorder suggesting shared neurophysiological mechanisms (Barrett, 1995). Interestingly also, 57% of patients were identified as having their alters presenting as dream characters in their dreams, and 26% as having at least two sub-personalities dreaming at the same time and experiencing each other as characters (Barrett, 1994), which not only gestures at the characters as a derivative of the person's integrated self but also echoes the several DMT participants herein who expressed a continuity between themselves and the encountered entities (Michael et al., 2021).

#### Experiences at “onset”

The following attempts to frame specific themes of the present analysis, including some subjective *content*, in terms of their possible neural substrates. For instance, one's perception of flowing through time has been indicated to be a constructed phenomenon within the generative model (Seth, 2021), where participants' sense of time dilation may derive from elevated prediction error signal and thus updating, as unpredicted stimuli have been shown to appear longer lasting (Pink-Hashkes et al., 2017). Medium microdoses of LSD also dilate time, even in the absence of subjective effects (Yanakieva et al., 2019). Descriptions of (open-eye) deconstructions or pixelations of the visual scene, prior to breakthrough elsewhere, is a vivid phenomenological expression of the attenuation of the precision of priors rendering the world-model fluid before the reversion of backward (top-down) to forward (bottom-up) traveling waves implying visual stimulation (Alamia et al., 2020), and alpha decrease and theta–delta increase underwriting sensorial memory (Timmermann et al., 2019).

#### “Bodily” experiences

The analgesic effect reported, including ecstasy, links to Timmermann et al. (2019) identification that the main phenomenological component of “bodily” changes during DMT correlated with reductions in the oscillatory beta band, and Timmermann (2019) fMRI results suggesting that the posterior operator network, implicated in pain regulation, shows disintegration. Two trials are underway to investigate psilocybin's effects on fibromyalgia (Gilligan, 2021; Hendricks, 2021)—and one case study of a psilocybin NDE-like experience also included near-complete fibromyalgia remission (Michael, 2022a). One participant reported perceiving themselves where they lay, where such OBE-like imagery is more expectable with ketamine (Luke, 2012). This may be owing to disinhibition or integration of the TPJ (as abovementioned, involved in the world-model construction of dreaming), neuropathology or stimulation of which has been shown to incite OBEs (Blanke et al., 2004, 2015) due to high-level predictive modeling of bodily/vestibular and visual experience becoming disconnected. The one experient, while sitting under a tree, demonstrating the herein-coined “phytanthropy” is reminiscent of altered states generated by *salvia divinorum*, in which the individual may feel themselves as transformed into nearby objects, or, indeed plant life (Luke, 2013). The disintegration of the DMN connector hub is not only present under the influence of DMT (Timmermann, 2019) but also salvia (Doss et al., 2020).

#### “Sensorial” experiences

The fluxing fractal geometry was stressed as being sentient in its own right by several, even equated with the experient themselves, that “it is *you*”, is discussed in Michael et al. (2021) and harkens back to similar evocations from Strassman (2001). This impression could be undergirded by the reversal of the anticorrelation between the DMN, mediating *internal* self-referential processes, and the task-positive network (TPN), active when engaging *external* attentional demands (Carhart-Harris et al., 2013; Palhano-Fontes et al., 2015). One novel proposition, too, of the reputed “hyperintelligence” (superior to the experient) of these visual forms and entities may be linked to the diminishment of confidence in predictive priors. This may then lead to a loss of experiential context for the raw sensory or intrinsic incoming data, where such a lack of prior standards as a reference point may result in the subjective sense of information associated with the imagery as unfamiliar and advanced—which may also help account for the reported noetic “insights” or “revelatory” states (possibly appearing mundane or unintelligible upon return), as well as the sense of “humility” in the context of ego diminishment.

While synaesthesia of some description was shared by many subjects (common to tryptamine psychedelics, especially LSD, and typically sound color, Luke et al., 2022), one individual, *ST*, depicted an elaborate multisensory-cognitive manifestation. Psychedelic synaesthesia may be prompted by the global hyperconnectivity of non-local networks, which is concomitant with the disintegration of the DMN, which facilitates ego consciousness. A very apt qualitative expression of both these processes was *ST*'s synaesthetic visualization of his body/physical internal state and a sense of self as a rotating hypercube and his utterance of being “sliced up into a million pieces”. Regarding the binding of the body and very selfhood in this image, the generation of the sense of self is itself likely to be another phenomenon constructed by high-level predictions, but fundamentally of interoceptive inputs from the body and viscera (Solms and Friston, 2018; Damasio, 2021; Seth, 2021). Whereas the TPJ, integrating audio-visual and body schema data, has been a “hot zone” for consciousness generation (Koch, 2019), *self*-consciousness may not arrive if not for its integration with the insula's interoceptive information processing (Damasio, 2021).

#### “Psychological” experiences

The very graphic instance in this study of “looping” is a classic example of this well-known part of psychedelic phenomenology. Such loops are a constant reactivation of the same intrinsically generated (autobiographic or imagined) percept or repeat stimulation of one's short-term eidetic or echoic memory of environmental stimuli. Balaet (2022) discussed the cognitive effects of psychedelics and reviewed psychedelic studies on memory. For instance, Williams et al. (2002) highlighted that 5-HT2A receptors have a key physiological role in working memory (such as in PFC) and that LSD alters neuronal networks implicated in memory (Kaelen et al., 2016). Not elaborated is that (in the context of music listening) this involved increased functional connectivity and information flow between the parahippocampus and the visual cortex, positively correlating with visual, including autobiographic imagery. Also discussed are impairments in spatial working memory (Wittmann et al., 2007), in lexical/numerical working memory, and decreased free recall (Family et al., 2020) under psychedelics.

The preservation of personal ego in several subjects ostensibly throughout their trips is interesting. This is in spite of DMT's resulting in DMN disintegration (Timmermann, 2019), as well as other psychedelics' effects of increasing global functional connectivity (Tagliazucchi et al., 2016), reducing parahippocampal-executive network and interhemispheric medial temporal lobe (mTL) connectivity (Lebedev et al., 2015); and reversal of anticorrelation between the salience network and DMN, or DMN and dorsal attention network (Stoliker et al., 2021)—all proposed as undergirding ego dissolution. However, it appears that the temporal dynamics of the DMT experience dictates a continuum with a sense of losing self at the peak (maximal disruption of neural substrates of selfhood) and a greater self-sense prior to and soon after this (concurrent with dualistic interactivity including entities).

#### “Emotional” experiences

Timmermann et al. (2019) have evidenced a correlation between the intensity of emotionality and neural entropy during the DMT experience, where such diversification of the neural repertoire is considered to index wealth of conscious content. This is consistent with the disintegration of neurally constraining high-level networks concomitant with the spontaneous release of subservient nodes such as the mTL (Carhart-Harris et al., 2014), housing the amygdala and hippocampal zones, limbic regions pivotal to memory and emotional regulation, and freeing of neural, psychodynamic, and energy (Carhart-Harris and Friston, 2010). This is phenomenologically reflective of the intense emotional “release” reported. The compelling feeling of “familiarity” also articulated (in ironic opposition to the humor evoked by surprise, maybe linked to undermined priors) likely pertains to neurobiological overlap with “déjà vu”. Those with temporal lobe epilepsy more prevalently report the sensation, where it is inducible *via* temporal cortical stimulation—and may also be owing to past dreaming of comparable material (Moulin, 2017). These findings are supported, respectively, by the disinhibition of the hippocampal structures (Carhart-Harris et al., 2014) implicated in memory encoding-consolidation-retrieval mechanisms and shared temporal slow-wave oscillations between the dream state and that of psychedelics and DMT (Carhart-Harris, 2007; Timmermann, 2019).

Finally, of the few severer challenging experiences, that of intense “fear of letting go”, and of accepting the death of one's own self as well as one's personal universe and all that it contains can be framed again in terms of DMT's disruption of the uncertainty minimization minimization by the cortical predictive processing mechanisms. This means the introduction of disconcerting chaos into the system. However, more specifically, the dissolution of the internal world model generated by the priors would be, as far as the individual experient is concerned, tantamount to the dissolution of the world itself, and thus subjectively felt as categorically apocalyptic. In addition, the “sense of dying” (at onset) reported to be associated with terror and breathlessness, including the “rush” effect betraying a sympathetic activation, may well be, in turn, associated with the speculated release of endogenous DMT at the moment of human biological death (and evidenced production during rodent cardiac arrest, Dean et al., 2019). Thus, exogenous use of the compound may trigger similar physiological cascades as that which any evolved endogenous release may cause in response to a threat to organismal preservation—or similarly, such use leads to the body's false interpretation of the DMT, including predictive attempts to explain its interoceptive effects, to be a signal of such a threat to life owing to its release as being physically correlated with the dying process (this is bolstered, e.g., in *EM'*s belief of her dying sensation is due to a feeling of “intoxication” by DMT, Michael et al., 2023, SSRN).

### Therapeutic potential

#### Experiences at “onset”

The powerful subjective features of the experience, in particular the emotional dimension, may prove important mediators of any clinical effectivity of DMT (e.g., Watts et al., 2017; Dos Santos et al., 2018). Even within the *onset* of the breakthrough, participants' experiences of abject “terror”, including “dying”, may serve as a powerful confrontation with one's mortality—a key tenet of existential therapy—where fear of death itself is suggested to be found many psychopathological conditions (Moreton et al., 2020). Continuing from such convictions of dying, “expansion” of the body and transcendence beyond its limiting boundaries, to find oneself in an ostensibly changed dimension, may also alleviate existential distress and thus improve wellbeing (Gandy, 2017).

#### “Bodily” experiences

Regarding *bodily* changes, such as some individuals noting distinct “vibratory” states, this is echoing of so-called “shaking therapy” or tension and trauma release exercises (TRE), inspired by instinctive neurogenic tremoring after traumatic events (Beattie and Berceli, 2021)—where at least one subject volunteered a feeling of trauma release during the vibrations.

#### “Sensorial” experiences

The account of *MS* found in the *sensorial* category, in which her skeletal imagery in rapid “flux” signified her existential impermanence, which, if understood, would result in humanity's betterment, points poetically to deep insights which can be garnered from the aesthetic content of the trip itself. Extrapolating meaning from symbolism and metaphor, such as that within the dream state but equally as legitimately in the psychedelic sphere, has been a cornerstone, especially of Jungian depth psychology and therapy (Hill, 2019). Though the hypercube was detailed by a number of other participants, the highly baroque “synaesthetic” episode of *ST* and the cube's inconceivable configurations seemed to emulate his very states of feelings and simultaneously served a progressive teaching function for him. Again, such unique displays within the experiential content itself may be considered a profound therapeutic technology. The experient's inner state, even selfhood, is vividly visualized and from which lessons may be extracted. This is not different from the gestalt therapeutic approach to dream states, wherein characters and objects are considered manifestations of fragments of the psyche (Alban and Groman, 1975).

#### “Psychological” experiences

The transient “memory loss” of one's prior condition and diminutions of sense of self, in the *psychological* domain, clearly rings of the egoless dimension of the mystical experience, well-documented to predict psychedelic therapeutic outcome (e.g., Haijen et al., 2018; Roseman et al., 2018). The flagrant descriptions by some experients of this cognitively disrupted, yet the productive state of mind, such as “coming from nothingness” and suddenly finding themselves in a given experience and flowing with it (e.g., *FF, SP*), is strongly suggestive of the meditative state—especially of mindfulness betraying a radical presence and acceptance of the moment. These are widely accepted to be crucial for mental wellness, their mirroring of central principles of acceptance-commitment therapy (ACT, Hayes and Wilson, 2017)—and its beneficial elevation also being identified after psychedelic experiences (Uthaug et al., 2019; Murphy-Beiner and Soar, 2020). In regard to the alternative, lesser reported preservation of the ego, one participant, *LG*, articulated an intense death-like scenario, but one in which his retained selfhood led him to conclude that “death isn't such a big deal”. This, akin to the discussion of DMT's existential implications above, points to how this feature, perhaps ironically opposite to the ego death, which prefaces therapeutic relief, also has the potential to relieve death angst and thus other related anxieties.

#### “Emotional” experiences—Positive

With respect to the *emotional* effects of DMT, the poignant experiences recounted by most participants, not least *BB, RV*, and *AF*, and others, maybe a testament to the psychotherapeutic potential of this unique psychedelic state. Indeed, an emotional breakthrough has been substantiated as a distinct component of psychedelic phenomenology (this paper, see Supplementary material 1), where its presence has also significantly predicted improved wellbeing, as did the mystical experience, which is well-known to do so (Roseman et al., 2019). To begin with the *positive* reactions, by far the most dominant feeling, the commonly felt “loving connectedness” was manifested in some experients' relating to the entities themselves, whereas for *BB*, it emanated between himself and his son's presence, which he effusively framed as a healing experience of his own inadequacies in love. *RV*, too, was movingly heart-opened, expressing an acute reversal of his hitherto “shutting-down” of his deeply felt aspirations to be a loving person, which he realized to be, in fact, a universal purpose—though also revealed, was the challenge of the inherent need to “let go” with such unification. This type of overwhelming reconnection with oneself and others is yet another pivotal subjective mediator of psychotherapeutic healing from the psychedelic experience (Watts et al., 2017; Carhart-Harris et al., 2018; Kettner et al., 2021; Roseman et al., 2021).

The feelings of immense “beauty” and “profundity” volunteered by very many, encapsulating the aesthetic sublimity and conceptual depth of a seemingly more-than-real alternative world, could be argued to have an ameliorating influence on the crisis of meaning that the contemporary world is presently facing (Cormier, 2018), which in turn addresses the same “givens of life” as those intrinsic to existential therapy (i.e., meaning, death, isolation, freedom, Yalom, 1980). The “humility” when confronted with the enormity of the DMT sphere that several participants disclosed and its mental health relevance is congruent with psychedelics' acute undermining of the ego. For *RV*, again, this crystalized into nothing short of a revelation of the hero's journey, a mission, though one of selfless service, in which he wished to “bring to humanity” the gnosis he received from the DMT. As such, the therapeutic reach of the experience can be seen not to be limited to the individual, but how their healing aspires to heal others. *BB*'s humbling encounter involved his insight into having asked “the wrong question”, replaced with the “correct” one—a psychological paradigm shift for him. Both experiences link to such a quality of insight in the psychedelic space being instrumental in the positive repercussions (Erritzoe et al., 2018; Davis et al., 2021), with this process being mediated by greater psychological flexibility (Davis et al., 2020b) like that during a reconfiguration to a less egoistic perspective.

In the subtheme pointing to DMT's “healing” properties, *BB* outrightly stated his unbidden revelation of the “cause of his suffering”, and *JA*, during his ritualistic sound-making, shared that he felt a reconnection to the divine replacing his everyday sicknesses. This latter is very evocative of the goals of transpersonal psychotherapy (TP), in which the dimensions greater than one's ego are worked with to enact healing of the person as a whole (Grof, 1973). The profuse feelings of “release” were alternately articulated as the compelling insight to “just *be*” (*AF*) or that “what is just is” (*SH*)—both redolent of the detachment and acceptance, and ayahuasca and 5MeO-DMT's mindfulness enhancements, discussed above in reference to *psychological* effects. These two participants (also *BB*) were, in addition, those whose experiences most characterized the so-called “Cosmic Game” (an entity communication identified in Michael et al., 2021)—expounded on with the following theme of “humour”. One technical theory of laughter is its derivation from a surprising or unpredicted stimulus, which is why it may be a classic reaction under psychedelics given their relaxing of “priors”, i.e., beliefs learned from experience enabling the prediction of our world—which, when relaxed, undermines the rigidity of psychological constructs contributing to psychopathology (Carhart-Harris, 2019; Carhart-Harris and Friston, 2019). Such deep-felt hilarity of the DMT trip is inherent to the idea of the “Cosmic Game”, a theme reflecting a realization of existence as being a “divine comedy” and the absurdist nature of the human condition, tantamount to the Hindu concept of *Lila* which denotes the fundamental playful purpose of the cosmos (Watts, 1989). The psychospiritually therapeutic potential of this dimension to DMT or psychedelic phenomenology, in its profound re-envisioning of the universe, may be hard to overestimate—in light of the *pronoia* essential to the experience, diametrically opposed to the paranoid stance to the world.

Several participants expressed their “gratitude” for the DMT experience itself and the profundity and healing it brought, self-evidencing the conduciveness of DMT to wellbeing. Such deep gratitude possesses a great capacity to generate a central perspectival shift from one of a sense of deprivation and emptiness to a sense of abundance and fullness. Indigenous societies, typically less psychopathological than contemporary ones, ritually give thanks for their provisions by their natural environment—where the modern world has turned to such techniques as “gratitude journaling”, empirically confirmed to bolster indices of mental health (Jans-Beken et al., 2020). Several participants also related the “familiarity” of the otherwise alien DMT world, including the entity characters therein. They couched this as making even the bizarreness or transpersonal events such as egoless unitivity as a comfortable or even perfectly natural process (e.g., *AN, FF*)—which would have important implications for any naïve and/or patient populations to be administered DMT, if medicalised, given the possible softening of its ontologically or otherwise shocking nature.

In considering all these desirable reports, it should be acknowledged that the specific participant population used here (self-selected, DMT experienced, psychedelic culturally engaged, as expounded in the Limitations section later), not least their lacking any clinical symptoms of psychiatric conditions including or often co-morbid with anxiety (Stein, 2001), such predominantly positive, and only scarcely frightening, DMT reports would have been predicted. Some challenges were evident still; for instance, the sheer “intensity” reported near-universally at the onset did entail a deeply felt threat to life and sometimes overwhelming feeling of instability of there being no experiential reference point—W. B. Yeats' resounding line that “the center cannot hold”—as part of the ego-quashing peak of the trip, could present a real psychological danger to any naïve/patient groups. That this same onsetting deluge was framed with watery symbolism is also reminiscent of the Joseph Campbell quote that “The psychotic drowns in the same waters in which the mystic swims with delight,” which may also imply a cautioning of those with any predispositions to psychotic experience. The other *neutral* theme was the demonstration of “letting go” during the experience, mentioned expressly by a few, but particularly *OR*. His deftness in navigating the sometimes chaotic scene, he claims, is a testament to the value of his religious framework, which he brought to the experience, and thus the usefulness in possessing training of some nature in conceptualizing and exposing oneself to the space (conversely to his atheistic DMT partner). This, importantly, is contrary to the present view reflected in psychedelic clinical trials, which are not hesitant to give, potentially ontologically shattering experiences with traditionally entheogenic substances, which classically give rise to mystical communion otherwise reserved for the mystic; the *mysterium tremendum* being one description to encode the awe simultaneous with the terror it may engender for the uninitiated.

#### “Emotional” experiences—Challenging

As per the *Challenging* experiences, though they were few and far between (amongst a DMT-experienced group), these still had harrowing qualities to them. *RV*, for instance, albeit the only one doing so, spoke painfully about his “fear of letting go”—starkly contrasted to *OR* above—wherein past traumas were reignited, which themselves then thwarted an ability of “ecstatic self-losing”. He accentuated that even if such self-loss were accomplished, it must unthinkably bring with it the dissolution of his personal universe, populated by his beloved family. This being one case, alongside others such as *RH*'s fractured void, *BW*'s psychotiform confusion, or *ZD'*s existential dread—these instances demonstrate the limit to which the human spirit can be stretched. Though none officially reported any clinically sustained distress, a couple still shared lingering difficulties that should be recognized by any DMT studies going forward. Finally, the death-like scenario painted by *LG*, interestingly not ego death, is a reminder of the capacity of DMT to elicit NDE-like episodes (DMT being the main ingredient of ayahuasca, “vine of the dead”), which are sometimes of negative valence and even lead to trauma-like reactions (Cassol et al., 2019)—albeit in *LG's* case, the traumatic reaction did not occur, he was in fact moved by the experience to appreciate life in its transience. Mirroring *RV*'s suffering at the prospect of his own annihilation concurring with losing loved ones, *LG's* experience of nightmarish “grief” comprised of a refusal, but acknowledged need, to accept his death and thus the “death” of his relationship with his girlfriend and her own inevitable grief.

For these challenging episodes, it is important to note that these never characterized the entirety of the trip. Positive components were also shared within these same reports (e.g., *RV, RH, LG*). Significantly, the vast majority of the experiences herein were benign and beneficial, despite the illicit and uncontrolled nature of the usage, which is claimed to elevate the probability of “bad trips” (Barrett et al., 2016), though perhaps less so among such seasoned users, Indeed, having specific recreational intentions has been shown to predict *less* challenging experiences (Haijen et al., 2018). Moreover, in spite of such unpredictability of the experience, especially with less experienced users, there are guidelines helping to moderate such experiences in controlled research that are applicable to future clinical practice (Johnson et al., 2008), including screening for contraindications, sufficient preparing and aftercare of participants, and curation of the setting, such as being in a safe space alongside trusted people.

Similarly, challenging experiences can be partially predicted and thus limited, where for instance, they are positively associated with neuroticism (Barrett et al., 2017). The degree of difficulty and harm has been predicted by the co-use of mood stabilizers and several variables related to set and setting (Simonsson et al., 2023). Whereas setting clear intentions predicted having a mystical experience, having a positive “set” reduced the chances of challenges (Haijen et al., 2018), and surrender also predicted mystical experiences (Russ et al., 2019).

In any case, distressing episodes may predict positive outcomes after the event. Their occurrence has been evidenced as being related to meaning, spiritual significance, and improvements in wellbeing (Barrett et al., 2016). Centrally, some psychedelic users choose to deny the term “bad trip”, emphasizing instead that for them, such experiences have mostly provided insights of a profound existential and transformative nature, where the authors conclude that this term can be thought of as a coping mechanism in the form of narrative sense-making to facilitate life-story integration, explaining why users continue to use even after such trips (Gashi et al., 2021).

This being so, it is vital that any employment of this potent substance is attentive to the reality of such challenges, whether sensorial, psychological, or perhaps especially (e.g., see *RV*) metaphysical or ontological DMT, indeed all classical psychedelics, are not only experiential but are considered by shamanic societies as *entheogens* (“generating a sense of the divine within”), and as such are unprecedented as potential medicines in the psychiatric pharmacopeia. It has been suggested that the new medical interest in these molecules represents the maneuvering of spirituality through the back door of science, an institution possibly not wholly equipped to resolve the psychedelic mystical experience, and which may be appropriating this practice of indigenous groups who have developed an acute understanding of the putative metaphysical implications and how to navigate any formidable consequences (Corbin, 2010). For instance, 18% of DMT users were found to have undergone “conversion” experiences, constituting a drop of almost two-thirds of those subscribing as “atheist” before encountering otherworldly entities on DMT to “non-atheist” afterward (Davis et al., 2020a), where such ontological shifts may have therapeutic potential [psychedelic mystical experience: Roseman et al., 2018; entity encounter: Lutkajtis, 2020; near-death experience (NDE): Van Lommel et al., 2001], and belief shifts post-psilocybin toward panpsychism correlated with improved mental health outcomes (Timmermann et al., 2021). Nevertheless, such metaphysically intense experiences may simultaneously be the driver of subsequent challenges, which often include ontological shock (mystical: Michael, 2022b; entity: Davis et al., 2020a; NDE: Pratte, 2021). For example, though small, one percent (26 respondents) of Davis et al.'s (2020a) DMT users endorsed that the experience provoked an “undesirable alteration in their conception of reality”, and some arguments suggest that materialism is a coping mechanism to defend against disturbing aspects of one's self and world (Kastrup, 2016), which if undermined may actually be deleterious.

The present study neither investigated the long-term effects of DMT nor explicitly seek to explore therapeutic applicability, though many participants did volunteer post-acute effects. These ranged from the simple and positive “I feel [relaxed] for several days afterwards” to much more ambivalently complex and perplexing experiences, which evoke the magnitude of the ontological shifts possible and yet emphasize the extreme cautionary stance one may have to take toward them:

“There's part of me that, a bit like in *the Matrix*…I almost feel not entirely right about taking people out of the Matrix unless they're absolutely called to it… should I give my wife the opportunity to see this? I don't know, not unless she's absolutely called to it, because it's *so* disorientating. Perhaps its[sic] better just to live in the Matrix, and try to find love and be love, find humility and be humility[sic], and try and live, try and be everything you can, without this extraordinary shamanic experience, which is *so* real… Every religious[sic]- *every* attempt at being spiritual or religious…is some attempt to connect with this. However misguided the religion…there's an attempt to try and wake us up to this extraordinary thing, and *truth*” (*RV*).

In relation to the therapeutic potential of DMT to treat depression, it is pertinent that initial findings of phase I and phase IIa clinical trials are promising (Baker-Jones and Campbell, 2022; Small Pharma, 2023).

## Limitations

The initial report on the DMT field study (Michael et al., 2021) elaborates in depth on the remaining limitations of the study, despite its addressing constraints of past research and the reader is again invited to refer to this. A summary here would include over-representation of Caucasian men (mostly White British, see Table 1), self-selection bias possibly predetermining the quality of the trip, stringent screening including past DMT experiences meaning non-naiveté and exposure to psychedelic culture, thus influencing experiential content (Hartogsohn, 2016), an untested plant-extracted physical substance used, and an uncontrolled naturalistic vs. lab environment. All such shortcomings being so, certain justifications can be made where, respectively, this demographic constitutes typical DMT users (Palamar and Le, 2018), ethical reasons contributed to using experienced users, experiences after use of the untested DMT are patently congruent with past research (see *Comparison with other DMT studies*), and lab contexts problematically introducing primes (Luke, 2017).

To elaborate on the self-selection of those with a positive attitude to DMT use and recruitment of, often very, experienced users (see Table 1) engaged with the psychedelic subculture, this would have certainly introduced a bias in terms of the resultant content of the DMT experiences, as psychological history (“set”) is a central driver of experiential content (Hartogsohn, 2016). Brief examples of this include Davis et al.'s (2020a) noting that respondents' most memorable entity encounter was during their first DMT experience and Strassman's (2001) finding that, of the high-dose participants, most of whom were initially naïve to DMT, around half reported such encounters, as compared to the present study's 94% prevalence of encounters. Other than this, it is not straightforward to discern how this specific population's DMT experiences would differ from a naïve or non-self-selected one, and it is beyond the scope of this one qualitative study to correlate the content of experience with the number of prior DMT experiences, which itself may be associated with influence by the psychedelic community. Future research could fruitfully address this relationship. However, peak intensity ratings for all participants (apart from one 7/10) were at least 8/10, indicating that the experience will be uniformly intense despite past experience. Also, while set and setting are well-known conditioners of the psychedelic state, such priming has not been empirically shown with DMT, and the use of such subjects was advantageous in terms of mitigating overwhelming reactions and optimizing phenomenological recall.

## Data availability statement

The raw data supporting the conclusions of this article will be made available by the authors, without undue reservation.

## Ethics statement

The studies involving human participants were reviewed and approved by University of Greenwich Research Ethics Committee (Ref. 17.3.5.15). The patients/participants provided their written informed consent to participate in this study.

## Author contributions

PM and DL: conceptualization, data curation, and funding acquisition. PM, DL, and OR: methodology. PM: formal analysis and writing the original draft. PM and OR: review and editing. DL and OR: supervision. All authors contributed to the article and approved the submitted version.
