# Supplementary material for: An encounter with the self: A thematic and content analysis of the DMT experience from a naturalistic field study
Source: Front Psychol. 2023 Mar 27;14:1083356. doi: 10.3389/fpsyg.2023.1083356 (PMC10083325; doi:10.3389/fpsyg.2023.1083356)
Supplement: Supplementary file 1 [file Data_Sheet_1.docx]

**Supplementary Materials**

**SM 1.**

**Meta-narratives: Co-creative Insight into Heart and Cosmos**

Juxtaposed to all previous discussion, the following themes pertain to a more interpretive function of participants’ narratives; their reflections on, as opposed to the content of, the experience in itself.

*Mind-manifestation*

Given DMT as a “psychedelic”, in the vast majority of cases, thirty, it was evident at some point that its *Mind-manifesting* nature – as is the meaning of the neologism – was being displayed. BB is extremely articulate in giving voice to this very dream-like effect of how the psyche seems to generate imagery inspired by one’s mental and emotional substrates, that is, the ‘set’ (17) of their mind prior to the experience – in his case the percolation of the notion of his child:

“it didn’t immediately feel like my son, it felt like someone in that role which fulfils that thing. So naturally my brain, just like a dream, puts a face to it…’And here’s one I made earlier!’…

this very, like I said, very strong sort of presence of this particular type of character, represents this particular type of thing to me – my son…

The reason I mentioned a dream, a dream-state, is because things the dream-state versus reality are kind of the opposite. So for example in reality, you see something immediately concrete and solid and definable, that over time affects you and changes your inner state, that ultimately ends up making you feel something. But in the *dream* and *psychedelic* state, it’s the exact same things but in reverse, just in the opposite order. So you feel something incredibly strong which gives rise to associations, which ends up potentially forming a visual image. But that image isn’t what it is, it’s just what you come up with…it’s like the word that best fits. So it wasn’t my son – ‘oh hey, hey Bean how’s it going’ – no, it was just wow, this form of love, that form of love, was just there, this type of relationship. Not even a person. Like I said, in the most abstract- in normal life you end up with feelings which are abstracted about something specific. And this started off in the complete abstract, so a form of love, which crystallised into – ‘oh, familiar, yeah I know that…!’”

‘Intentions’ set by participants, or more commonly, ‘personal actions’ that they themselves were enacting within their experience (11), included, as per the former, SP (Trip 2) who “went into the experience basically saying, giving myself to the universe” going on to, indeed, experience a participation in the creation of the cosmos. As regards the in-trip functions of the psychonauts – explorers of psyche – most disclosed they were “just witnessing it”, serving as “only a visitor, an observer”, while BB, having explained his “sensed presence of pure love” as specifically played out by his son, elaborates that there was also “[an]other one, the sort of, its protector” as he himself “was filling that role as well, the…unconditional protective love”. One of the more purposeful and proactive participants, OR’s (Trip 2) attempts to journey with and aid his struggling DMT partner were inspired by his Barquinha religious imperative:

“I felt like there was an indigenous guy with me who was strong… And there was a tone I needed to *Whoof*, you know this is the sound we make when we are with spirits in Barquinha, and I felt like *Whoof* I was helping him to clean some of these companies [i.e. spirits]... this happens sometimes when I’m working with Pretos Velhos, and disciplining souls

Interviewer: Disciplining souls?

Yeah, imagine in Brazil you see tricksters or souls like this, and I felt like when I tried to concentrate and put energy into separate… *wheat from the chaff*, like something like this… he was having a, from my interpretation, a bad trip. And then again, I started to try to connect with him…in Barquinha we would say the interpretation is there are some lost souls there, some materialistic point of view, some arrogant…companies… imagine you had a dense cargo, so I get him in, I take from you or from the other, I took from you and send [it] to another realm, you know…

I felt connected with him spiritually…I felt compassion for him… I feel like I have responsibilities to do what I have learnt… if I feel he’s in trouble, I’m trying to connect myself with light, and maybe if I may if it’s possible to help him… for me…it was like he was having a spiritual attack”

The following is comparable to much of the above, but specific in terms of the subjects’ deliberate employment of their attentional focus within, and its direct influence upon the experience – as contrasted to the, thus far, vastly passive experiences. This ‘co-creation’ dynamic is particularly of note, mimicking the level of control one has when commanding a ‘lucid dream’ (10). To begin with, all AZ, RS and LR mentioned, respectively, that “geometrical figures were multiplying and I could somehow manipulate them”, “the [Aztec] patters…sometime you can interact, and you can move them”, “I was trying to manipulate the visuals…and push them along”. HV discusses something reminiscent of shamanic practices to engender her own physical healing by similar visionary manipulation:

“Like a whirlpool…a smoky whirlpool... So that’s why I was using the wands, to unwind it and unravel it because it needed unravelling… a whirlpool of sickness, so I was unravelling it, unravelling it…then it just started becoming like a part of the atmosphere… it was something that was just clogged up and needed to be untangled, I was untangling it…

that was the bit when I was cleaning myself out… that’s the bit I was like ‘how come it’s not in my leg?’ I thought it was gonna be in my leg, but it was in my back – then it was OK”

Both RH’s (Trip 1) and SH’s amorphous environments, likened to a “building” with “rooms”, responded to their mental effort in moving forward. For example:

“I have this mantra going, that whatever is there – ‘Concentrate deeply, concentrate deeply’…and that’s when all hell breaks loose, that’s when it all fractured…loads of dimensions everywhere” (RH 1)

Most illustrative of this participatory nature of the DMT reality, where concerted attention plays into experiential construction, includes AF’s report – which like SH’s, emphasises the function of *movement* as one engages with the world, and both comprising an annunciation of the *Cosmic Game* (the world as engineered to play and experiment with):

“And this was showing me there's just so many other ways of looking at things, if you put your attention. And again…this thing [multidimensional cube] was moving, because I was looking at them, wherever I was looking, putting my attention, it was *becoming*. I put my attention there and these things are ready to move, put my attention there and these things are ready to move. So these sensual females, they were like…’It depends where you’re looking at things and how you’re looking at things!’ …they were telling me that everything I put my attention on can start moving, everything I want is possible – I just have to put my focus into it, and the game will start. But I have to keep moving. Life exists in the movement, and everything moves” (AF)

Importantly, while AF’s lesson in the power of attention seems exhibited by her hypercubic object, so is this replicated in BB’s description of his frenetic cube which:

“rotates, sort of spins, not slowly…almost as if it’s trying to catch your attention, basically, as if it’s trying to spin and add extra layers of colours and detail just to grab you, and in front of your eyes it does this. If you stop paying attention to it…it’s off on the side. But when you look at it, it moulds in front of you. The act of looking at it, paying attention to it, *creates* it” (BB)

*Ontological and Emotional breakthrough*

Inter-spliced with the experiential narratives, the predominant focus of the interview, most subjects, twenty-seven cited here, would naturally voice their opinions on the ontological status of what they had just gone through, its consequences for their worldview, and the deep learning they would take away – thus all encompassed under *Ontological and Emotional breakthrough.* First, subjects’ ‘metaphysical beliefs’ (18), evinced by the felt ramifications of the trip, were often profound, many of which asserting its hyper-real, that is *more real* than real, nature. MP states that although his scene, with which he felt “a merging of my body” in a “non-dual relationship”, appeared “super holographic, very digital looking”, it was also “hyper-real”, elaborating that he enjoyed “Knowing you’re immortal, that there’s no death, knowing there’s much more to reality than just the physical”. Articulated by LR again, the DMT space was “So real, so real. So very real. If anything, it was like, realer than real. Such a cliché”, he eloquently builds the model that:

“this is my essence and this is the true form of all things, and everything that I am when I’m not paying attention to the true form of all things is kind of like, it’s like *that’s* the dream, that’s the distraction from the reality, and *this* is the reality that I’m in”

RH (Trip 2) labours this idea yet again, saying it is “more real than real, I know it’s a cliché, but just so fucking real!”, and that typically there ensues a progression from “geometry and fractalization” toward ultra-vivid iconic scenery, or “very solid, well if it was a film, a very, very well-conceived film”. Proclaiming upon return his “110%” credulity, “There is no question. Fuck me I am so, so convinced” he appeals to deeper versus farther worlds, that it may be “a quantum world we’re going into”. This sentiment of the inter-relevancies of “deep physics, metaphysics” and the DMT trip is shared by RS, claiming that the entoptics are “in this world, regardless of if we see them or not they’re there…I really do think there’s something to it with dark matter”. Such ideas he develops slightly by “the idea of consciousness not generated by the brain, but antennas picking up certain frequencies – it’s like the DMT tweaks the antenna a bit”, which in turn, returning again, foreshadows RH’s statement of a similar frequency that it’s “Almost as if [the entities] are having a similar experience… I do often wonder – does DMT not only enable us to break up some sort of inhibiting thing of the mind, but…does it allow them to see into our experience too?”

In RH’s third experience, revelations of such incorporation on the part of the entities (see *Active involvement,* possession), are first prefaced by him with – still yet re-incorporated into consensus reality – “Wow. It’s like you’re so definitely here aren’t you [i.e. the entities]. Oh my God, you’re definitely here”, He further narrates a vivid metaphysical model that:

“it’s two levels, like the human inside of you, ‘my brain’ human side of you- …The human side had some surprise and some shock at all of this. But there’s a part of me that just knew it all…it knew what to do. It knew what to do! Oh God, I’m not gonna censor myself, it honestly feels like: It’s a Soul that knows what to do out there, whereas the brain, it’s kind of useful for putting it into words that makes sense down here, but it feels like the soul knows exactly what to do… Like it has another *life* even, like it has a *completely* other life. I often feel like it’s simultaneously existent in another plane whilst I’m partly connected to it in this experience…

We’re nothing like we think we are! It’s just beyond man! [Laughter]

Interviewer: We’re not who we think we are – in what way?

We think that we are solely in this material, 3D/4D world. But I’m absolutely convinced we’re doing this all the time. These entities, they’re right here!... I mean, I cannot be shaken from this conviction that it’s completely real, it’s really, really happening, right next to this existence, another realm, right here…

[These entities have] a huge desire to experience somehow, and that’s why I was a bit baffled when I came out, why I’m not quite human – …the universe wants to experience itself, and it gets better at it with DMT. It’s not the universe, I wanna get detailed about this: It was these creatures, these entities wanted, really want to experience everything… Then I’m not convinced I wasn’t one of them”

Seconding RH’s imminence of this other world, ST adds, alongside its insight-imparting qualities, that it “seems to be a thing where I step into this realm, and this realm is always here, and I step into this realm and this is your lesson for today. It’s an endless class, every time you come there’s always a lesson”, again alluding to reminiscences of quantum physics, with his shifting hypercube constituting “a fundamental thing in reality…the Planck scale”. Words of conviction about the more intrinsic depth to these far out worlds are similarly also oftentimes threaded with the suggestion of connection to, and implications for the nature of, one’s Self, as just clearly explored by RH, as well as by ST in another elaborate explication of his hypercube as being, essentially, “me”. In this vein, OR (Trip 1) also remarks that “it’s so incredible how the spaces inside me actually gain another kind of space. It’s like you can actually, *Ooh*, open this thin part of reality and fill that with images…and consciousness”. Again, OR brings up the repeated framework that, “It was like I was channelling to that frequency and that’s what was happening on that frequency”, and when asked as to the felt ontology of his “Preto Velho” spirit guide, he echoes this self-other consonance and dissonance:

“I would say another consciousness, separate to me. Imagine, actually it’s the truth, but imagine my thoughts are in a wave, but when I feel this presence, I can realise the *shift* of these waves. So it’s like even if that *is* me, I realise it’s another part of me that thinks and realises and sees reality in a different way to me. Like I can actually be like, what I understand as [OR] thinks this way, and these other thoughts, I can have this feeling of I agree with you, but somehow I feel it’s not me”

The reality-status of the entities is discussed along very similar lines by JA, again in the form of spirit guides, but more assuredly other:

“It’s happened too many times…I feel like I’m in dialogue with something *outside* of myself because I’m being told things I just don’t know… I’m given teachings or understandings of things I don’t think I could come up with myself. And the dialogue is with such a different quality to ones I have with myself as well”

The cosmic wool as constitutively pulled over our everyday eyes, and the scales falling from them upon DMT intoxication – with very “real world” repercussions – is further championed by MS:

“when I had my eyes opened before and today, that's when I felt the connection with everything, the breakdown of what you see is not what is real… it’s just a feeling of mine…just how we perceive everything so wrong really, I suppose. And with that, it’s showing you what *really* is there, sort of thing. Bizarre. Very bizarre…

It’s almost like we really live in an illusion in this world of ours, and people need to wake up and believe in other shit!... You know, for the sake of the- *humanity*, not the planet, because the planet will survive because the planet knows what it’s doing”

The unmitigatedly extant, as well as paradoxically estranged and imminently embracing, nature of the DMT realm is again reaffirmed by RV:

“when I was presented with that space there wasn’t even, there wasn’t even the tiniest sliver of doubt as to its ultimate reality, as to the fact that somehow, somehow it exists, you know within, beyond, contains you know, this world”

Soon after inciting “the truth of this and to [that] reality” and his desire of “bringing that truth to humanity”, RV then provides a development, more a profound soliloquy than participant response, of this matter of breakthrough epiphanies of such sublimely real spiritual realms against the backdrop of comfortable illusion, and human beings’ burden of will upon their revelation:

“it’s so disorientating that you almost don’t- you know there’s part of me that, a bit like in the Matrix, I’m always- I almost feel not entirely right about taking people out of the Matrix unless they’re absolutely called to it, you know what I mean? Like should I give my wife the opportunity to see this? I don’t know, not unless she’s absolutely called to it, because it’s so disorientating. Perhaps it’s better just to *live* in the Matrix, and try to find Love and be Love, find humility and be humility, and try and live, try and be everything you can, without this extraordinary shamanic experience, which is *so* real, you know. Everything, it is *so* real, you know. Every religious, every attempt at being spiritual or religious, every attempt at being spiritual or religious is some attempt to connect with this. However misguided the religion, however- there’s an attempt to try and wake us up to this extraordinary thing, and truth”

In a crucial turn, in terms of illustratively juxtaposing participants’ reflexive ontologies, while RV evokes vivid parallels between the *Matrix* saga and his own gnostic deprogramming, BW here refers to his last DMT experience as very similar – in which he viewed his world and loved ones as composed of computer code – yet, his current trip in fact reversed such beliefs in some celestial simulation:

“I came out of that last [DMT trip] for weeks still mildly psychotic, it lasted for weeks and we talked about it, ‘the Matrix’. Like is there a matrix, are we- ?

Interviewer: How do you feel about it now?

I don’t feel it at all, not at all, no hint of that at all. No hint of ‘I've glimpsed what the world is really like!’ [Last time] I came out of it like I believed I glimpse the true nature of reality. What I just saw [today] was not the true nature of reality, *this* [consensus world] is the true nature of reality

Interviewer: And how does that make you feel about your previous DMT matrix experience?

In some ways relieved, and in some ways disappointed! Disappointed that it hasn’t shifted me existentially like the last one did. I feel frighteningly, boringly *not* existentially altered by it. It was a mind-blowing *drug* experience. But I came out of the last one talking for weeks about ‘is it real or not!?’, and now I just know it was a drug experience”

As firmly evidenced, DMT’s baffling world necessarily calls one to question the ultimate nature of things, confronted with its deeply sensed reality despite seeming so, apparently, other, and starkly unlike what one is accustomed to in waking life. This ‘death by astonishment’, as paraphrased from the DMT promulgator, Terence McKenna, resounds with many participants’ comments already on the sheer intensity of the trip, but is further explored here around the ‘ontological shock’ (12) by the deconstruction of their prior world-models.

Both GR and TC echo one another, the former exclaiming, “it just kind of exploded into something… everything was like completely shifted into another way of interpreting reality”, and the latter, that “I could feel and see all my current issues kind of wrapped, and being completely shaken and… What I think what is reality all of a sudden got completely questioned”. The inner structures maintaining the daily rhythms are disintegrated, as in DD’s words:

“I was like ‘I’m fucking up for this’ – I found that made it definitely easier for me to transition- but that *shredding* of perception…

Interviewer: Like you were saying before the trip with me, that switch of consciousness?

Yeah, yeah exactly, It’s almost like the inner working of the brain that has to deal with linear time and your life on a day to day basis, going *Vrrrrrr* you know, *Ssshhhttff* like Dorothy [in the tornado]!”

Referencing his three trips, a part of each of them involving sensations of dying, usually in a “limbo-land”, RH reiterates this unprecedented ontoseismic calamity:

“And the terror… it’s more to do with the fact that reality is just so, broken, it’s gone. Where I am in that waiting room is just so not anything I’ve ever experienced” (RH 1)

“I’ve heard myself saying that in the trips before, just Wow or Fuck or you know, just trying to get my head around the extraordinariness of it

Interviewer: Did you know what *anything* was when you felt like you were dying?

No. This time definitely not, no… in the fracturedness I know absolutely nothing. That was a terrifying one…this one I just did, terrifying. God, I maybe thought I’d broken reality” (RH 2)

“Can you describe [the entities]?

Oh My God, no! Fuck it! It’s just too mind-blowing. It’s kind of not fair… To explain it was extraordinarily difficult…it just seemed so far removed from anything – even though I can now tell you that they’re boxes” (RH 3)

ST paints his hypercubic configuration in a symbolic light, where any attempt by his brain to predict its beguiling activity, an extension of his own mind, was futile – which is an insightful representation of life itself:

“it’s saying, ‘Imagine this [cube] represents you’…such that whatever is happening to it I am feeling… So it seems my set, so my expectation of what the experience should be, was totally blown out of the water by what I was seeing, and what I was seeing represented something that would be painful to that set of expectations…

And not just expect you’ll understand the patterns… you’re looking at patterns, your brain predicts what this thing’s supposed to do next…but this is showing you ‘Look mate, you cannot predict because this one has multidimensional directions it can go to that you can’t even fathom. Like ‘Oh that's possible!?’…you think it’s just up and down, and it goes inside out and sideways at the same time, and you’re like How the fuck, what!!?…

For me it just seemed…being always perplexed by what actually happens in life to me…I can relate it to, the way I expect things to go compared to the way they appear. Things can go anywhere man, from zero, there’s nothing to expect because look at what makes them up – There’s no predictable thing here, so the only predictable thing is this chaos!”

To finally reiterate this simultaneity of the experience’s interstellar yet deeply inter-fused quality, in revisiting FF’s “phenomenally alien, but familiar” world – although he admits that “except for my previous DMT experiences, there is *nothing* comparable” to that “quite terrifying, mind-blowing environment” – he still reinstates that “it’s somewhere deep down you know it, it’s familiar”.

Distinguished from noetic episodes of sudden comprehensions of everything, or messages communicated by entities, ‘insights’ or ‘breakthroughs’ (14) both in a more metaphysical and personal sense were equally profound. First, a revelation of “the love I already have” was already discussed by BB – a sudden, hilarious and salutatory realisation granted to him as part of the “*You Dick! moment”* of his ‘cosmic giggle’, which he completes with a modest “I’ve kind of got something really positive from the experience”. RV’s more severe, aforementioned cosmic disclosure of “the truth…of that reality”, paralleled to “taking people out of the Matrix”, was also adorned with such meditations on love – that “perhaps it’s better just to live in the Matrix, and try to find Love and be Love, find humility and be humility”, and a conviction in the discernment of the Buddhist aspiration toward a “very special kind of non-attachment which is about the heart… this unconditional love for all things”.

LR indicates a “reconfirming, just like, reminders of what I already know, this ancient knowledge within myself”, that is, an oasis of original knowledge available, but often inaccessible, to mankind – and reaccessible during such experiences, agreed by RS:

“we’ve definitely lost something, as humanity, we’ve forgotten something – we're definitely coming back to something, but we’ve definitely lost something of this old, deep realm

Interviewer: And you think this realm is as it appears to be, like full of other sentient beings?

Yeah, I think if we can communicate better with them, I think it’s really about us listening, and a big part of it is humans have forgotten how to listen to the world…we don’t realise that we’re part of nature and everything we make is also part of nature. It’s because of that problem, the separation, that things [are] out of harmony. So yeah, I’ve had presences being like this *symbiosis* before – we need to work with what else is here”

Resonating with this understanding of an urgency for a re-enchantment with the natural world (also touched on by MS, earlier), is AV’s statement that she “felt the responsibility that we have as human beings, to hold the space for each other, to look after the planet”. A different epiphany as to the natural order of things, for LG, was instigated by a dramatic plunge into a harrowing death scene, though embedded with lessons in gratitude:

“I’d had a fucking heart attack, and…everything’s been going so well between her and I, and I was like ‘Fuck this is it, she’s gonna be devastated’. But then…I was going ‘No, No, but this is the nature of reality’. This feeling, that this is the nature of reality, and it’s not always cushty, you don’t know what’s the round the corner, so saver whatever is going on at any point!...

all these thoughts came in like ‘This is the nature of how energy works – it’s gonna revert back on itself at some point’… how everything is transient, you go through these phases that’s neither one or the other, and death is just part of that process… It’s all good for a period of time, then you just get slammed with something fucking heavy, which is the way it is. You kind of forget about it when you’re caught into whatever cycle is going on”

In a much more reified exemplar of learning via the experience, ST outlines the intricate interaction he has with his hypercube-*cum*-himself, that he likens here, aptly, to a “brain-game that you play”, as if some psychospiritual rubix cube:

“So it’s like a lesson…and it teaches you… [it] shows you a different state, and you play to different stages and different difficulty levels… it would show me different levels of that which would automatically put me physically in a certain state which is new to me, because I’m a newbie in this game, at ‘high difficulty’ level…

it’s like a new language: *Associate this feeling with this pattern of movement*… the real thing that's the message – You were taught with 3 colours, there’s like 12 different colours and they were moving before in 2 dimensions, but now they’re moving in all different dimensions possible which is probably 11! So it’s like a programming as well, like my physical responses have been programmed to go with whatever pattern was displayed…this ‘high grade’ pattern”

He continues that, “this seemed like something I was teaching myself or something, or those beings were”, and alongside the cube’s (aforementioned) association with tripping over his own breath, upon letting go he realises what meaning to ultimately glean:

“‘Ok surrender to this’… surrender to whatever’s happening, just to breathe and let it happen by itself… breathe as you know how to breathe, you know, which is just live as you know how to live… Just live, live!...

So that generally speaks to me in saying, I can’t have expectations about people, or things, or situations, because these things must have a life of their own so to speak… If you think about breathing too much, like that demonstrated, you could die!... I tend to overthink things... Almost like you wanna be perfect before you make a move, like *bust* a move, everyone’s allowed to make mistakes, you don’t have to be an expert, that's how you learn… Breathing is basic, and we take it for granted even though it can be a problem, so why don’t I extend that to other things that I do – I can just do them, without having to check how I’m standing morally or this or that ticking all the boxes before I make a move, you know”

It’s particularly noteworthy that ST’s sentiments of relegations of over-anxious concerns or pressures to be morally immaculate, and protestations to simply *live*, as also potentially conveyed by beings, are all highly reminiscent of both SH and AF’s insights throughout their ‘cosmic game’ encounters (see **Encounters,** *Communication*) – during which the latter also witnessed a colourful, shifting “multidimensional cube”.

**SM 2.**

**Table 3. Table of all super-ordinate, mid-level and subthemes**

| **Encounter with the Self** |  |  |
| --- | --- | --- |
|  | Clarificatory notes | No. Interviews /36 (%) |
| ***Onset*** |  |  |
| *Sensory* |  | *14 (39)* |
| Submergence |  | 9 (25) |
| Geometry or Patterns (Onset) |  | 6 (17) |
| *Emotion & Body* |  | *18 (40)* |
| Laboured breathing |  | 7 (19) |
| Terror or Panic |  | 6 (17) |
| 'The rush' |  | 5 (14) |
| Anxiety or Fear (Onset) |  | 4 (11) |
| Pain / Torture (Onset) |  | 2 (6) |
| Trapped / Powerless |  | 1 (3) |
| Body expanding / melting |  | 1 (3) |
| *Space-time shifts* |  | *10 (28)* |
| Time dilation (Onset) |  | 6 (17) |
| Reality breaking down (Open eye; Onset) |  | 4 (11) |
| Immediately elsewhere |  | 4 (11) |
|  |  |  |
| ***Bodily*** |  |  |
| *Pleasurable* |  | *10 (28)* |
| Ecstasy |  | 4 (11) |
| Pain relief |  | 2 (6) |
| Fusion (with partner) |  | 2 (6) |
| Rooted |  | 1 (3) |
| Stretching urge |  | 1 (3) |
| Warmth |  | 1 (3) |
| Post-orgasmic state |  | 1 (3) |
| *Neutral / Both* |  | *8 (22)* |
| Religious hand-signs |  | 3 (8) |
| Vibrating or (Subjective) Convulsion |  | 3 (8) |
| ‘Phytanthropy’ | Subject’s transformation into a tree | 1 (3) |
| Falling sensation |  | 1 (3) |
| *Uncomfortable* |  | *9 (25)* |
| Heaviness |  | 1 (3) |
| Paralysis (Subjective) |  | 1 (3) |
| Sensitivity |  | 1 (3) |
|  |  |  |
| ***Emotional*** |  |  |
| *Positive* |  | *34 (94)* |
| Peace / Pleasantness |  | 16 (44) |
| Profundity & Beauty |  | 14 (39) |
| Familiarity |  | 11 (31) |
| Loving or Connected |  | 10 (28) |
| Gratefulness |  | 8 (22) |
| Humour & Hilarity |  | 7 (19) |
| Healing |  | 5 (14) |
| Release or Relief |  | 4 (11) |
| Humility |  | 3 (8) |
| Gentleness |  | 2 (6) |
| Womb-like |  | 1 (3) |
| *Neither / Both* |  | *21 (58)* |
| Extreme intensity, or Overwhelm |  | 21 (58) |
| Letting go / Detachment |  | 3 (8) |
| Wash of emotion |  | 2 (6) |
| Ambivalence |  | 1 (3) |
| Infantile regression | Clinging to, and biting their present partner, in a foetal-like position to feel safe | 1 (3) |
| Disappointment |  | 1 (3) |
| *Challenging* |  | *7 (19)* |
| Anxiety or Fear (During) |  | 3 (8) |
| Confusion |  | 2 (6) |
| Fear of letting go |  | 1 (3) |
| Grief (from own death) |  | 1 (3) |
| Guilt |  | 1 (3) |
| Pain / Torture (During) |  | 1 (3) |
| Personal struggles |  | 1 (3) |
| Traumatic re-experience |  | 1 (3) |
|  |  |  |
| ***Sensorial*** |  |  |
| *Open-eye* |  | *11 (31)* |
| Other open-eye visuals |  | 7 (19) |
| Reality breaking down or Pixilation (During) |  | 3 (8) |
| Clairvoyant-like |  | 2 (6) |
| Energy flow |  | 1 (3) |
| Skulls |  | 1 (3) |
| Depth-perception |  | 1 (3) |
| *Visual* |  | *27 (75)* |
| Geometry (During) |  | 16 (44) |
| Colourfulness |  | 13 (36) |
| Flux |  | 13 (36) |
| Fractals |  | 11 (31) |
| Hyperdimensionality |  | 8 (22) |
| *Cross-modal & Other* |  | *14 (39)* |
| Synaesthesia | Audio-visual (3)  Somatic-visual (3)  Audio-somatic (1)  Somatic-visual-audio (1)  Visual-cognitive-affective (1)  Somatic-visual-cognitive-affective (1) | 10 (28) |
| Sound (During) | e.g. Tones, Glitches | 4 (11) |
| Sensitivity |  | 3 (8) |
| Audio slowing |  | 1 (3) |
| Tactile | e.g. Walked on | 1 (3) |
|  |  |  |
| ***Psychological*** |  |  |
| *Memory & Language* |  | *31 (86)* |
| Difficulty expressing / Recourse to metaphor |  | 19 (53) |
| Temporary memory loss (within experience) | Inability to remember prior condition of ‘being in an experiment, to answer ‘intensity rating’ questions | 15 (42) |
| Partial recall loss (after experience) |  | 12 (33) |
| Looping & Intrusive thought |  | 4 (11) |
| ‘Schizophrenia’-like | Involving confusion between thoughts and speech, and sense of losing one’s mind | 1 (3) |
| *Awareness & Sense of Self* |  | *18 (50)* |
| Aware of surroundings |  | 9 (25) |
| Unaware of surroundings |  | 8 (22) |
| Lucidity / Ego preservation |  | 5 (14) |
| Clear mind |  | 1 (3) |
| Intuition |  | 1 (3) |
| *Time distortions* |  | *13 (36)* |
| Time dilation (During) |  | 5 (14) |
| Time contraction |  | 4 (11) |
| Other distortions |  | 3 (8) |
|  |  |  |
| ***Meta-Narratives*** |  |  |
| *Mind-Manifestation* |  | *30 (83)* |
| Influence of 'Set' |  | 17 (47) |
| Influence of 'Setting' |  | 16 (44) |
| Personal actions & Intention |  | 11 (31) |
| Co-creation / Lucid dream-like |  | 10 (28) |
| *Ontological and Emotional breakthrough* |  | *27 (75)* |
| Metaphysical beliefs |  | 18 (50) |
| Insight / Breakthrough |  | 14 (39) |
| Ontological shock / 'Death by astonishment' | Ontoseismic, or ‘world-shattering’, experience, given experience’s baffling nature or disassembling of all prior conceptions | 12 (33) |
| *Transitions through time* |  | *20 (56)* |
| Internal dynamics |  | 13 (36) |
| Repeated content | Experienced in previous DMT trips also (May be due to state-dependent memory) | 11 (31) |
| Novel content |  | 6 (17) |

**SM 3.**

**Graphic of all super-ordinate and mid-level themes – including those of *Encounter with the Other* (Michael et al., 2021)**


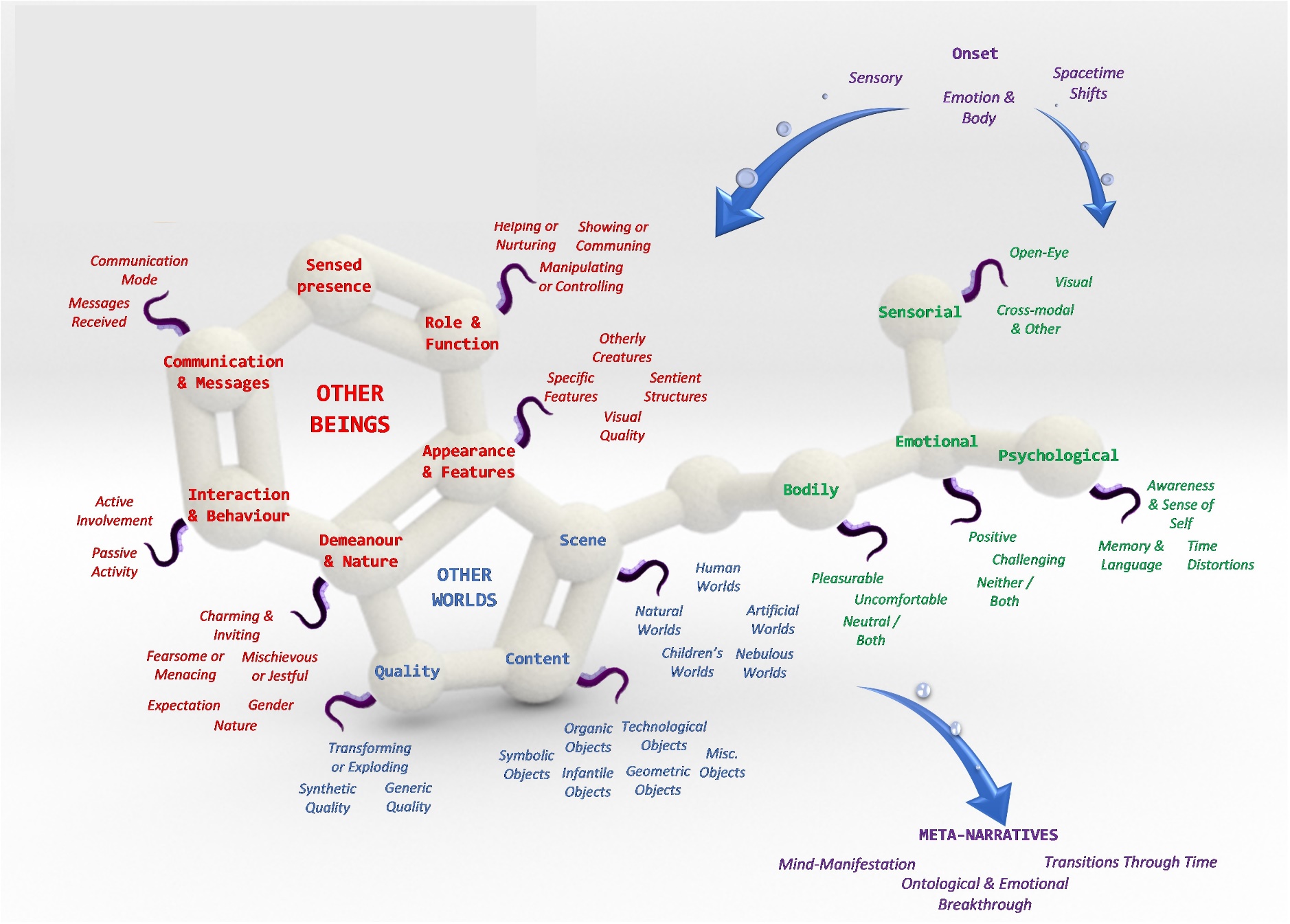


**SM 4.**

**Indicative Questions of Semi-Structured Interview (with initial prompts)**

- Please can you describe your DMT experience as best and fully as you can, and as chronologically as you can. Please use all your own words, and try to break down your experience into its detailed elements, while avoiding using terms and concepts from other people or popular culture

- What is the first thing you remember?

- How did your body feel during the experience?

How did your awareness of your body change?

- Did you see any geometric, fractal or entoptic patterns?

Where they colourful, or moving? What was their dimensionality?

- Where did you ‘go’ in your experience?

What did the scene look like?

- Did you have any encounters with other beings in your experience?

What did they look like? What was their approach toward you? Did they communicate with you? Did they interact with you?

- Did you receive any information, or have any particular insights or understanding?

- How was your sense of time affected during the experience?

How long did the experience seem to last? (Versus how long it *did* last)

- How pleasurable was the experience?

What other emotions did you have?

- What was your sense of self like?

In relation to the world around you?

- On a scale from 1-10 where 10 is the most intense and 1 is normal intensity of experience,

how intense did your experience get?
